# Prospective and challenges of locally applied repurposed pharmaceuticals for periodontal tissue regeneration

**DOI:** 10.3389/fbioe.2024.1400472

**Published:** 2024-11-13

**Authors:** Mohammad El-Nablaway, Fatema Rashed, Ehab S. Taher, Ahmed Abdeen, Noha Taymour, Magdalen M. Soliman, Hany K. Shalaby, Liana Fericean, Bănățean-Dunea Ioan, Mohamed El-Sherbiny, Elturabi Ebrahim, Afaf Abdelkader, Mohamed Abdo, Cucui-Cozma Alexandru, Gamal A. Atia

**Affiliations:** ^1^ Department of Basic Medical Sciences, College of Medicine, AlMaarefa University, Diriyah, Saudi Arabia; ^2^ Department of Medical Biochemistry, Faculty of Medicine, Mansoura University, Mansoura, Egypt; ^3^ Department of Basic Medical and Dental Sciences, Faculty of Dentistry, Zarqa University, Zarqa, Jordan; ^4^ Department of Forensic Medicine and Toxicology, Faculty of Veterinary Medicine, Benha University, Toukh, Egypt; ^5^ Department of Substitutive Dental Sciences, College of Dentistry, Imam Abdulrahman Bin Faisal University, Dammam, Saudi Arabia; ^6^ Department of Oral Medicine, Periodontology, and Diagnosis, Faculty of Dentistry, Badr University, Badr City, Egypt; ^7^ Department of Oral Medicine, Periodontology and Oral Diagnosis, Faculty of Dentistry, Suez University, Suez, Egypt; ^8^ Department of Biology and Plant Protection, Faculty of Agriculture, University of Life Sciences “King Michael I” from Timișoara, Timișoara, Romania; ^9^ Department of Medical Surgical Nursing, Nursing College, Prince Sattam Bin Abdulaziz University, Al-Kharj, Saudi Arabia; ^10^ Department of Forensic Medicine and Clinical Toxicology, Faculty of Medicine, Benha University, Benha, Egypt; ^11^ Department of Animal Histology and Anatomy, School of Veterinary Medicine, Badr University in Cairo (BUC), Badr City, Egypt; ^12^ Department of Anatomy and Embryology, Faculty Veterinary Medicine, University of Sadat City, Sadat City, Egypt; ^13^ Second Department of Surgery Victor Babeș, University of Medicine and Pharmacy Timisoara, Timisoara, Romania; ^14^ Department of Oral Medicine, Periodontology, and Diagnosis, Faculty of Dentistry, Suez Canal University, Ismailia, Egypt

**Keywords:** periodontitis, biological regulation, biomaterials, bioengineering, tissue remodeling

## Abstract

Periodontitis is a persistent inflammatory condition that causes periodontal ligament degradation, periodontal pocket development, and alveolar bone destruction, all of which lead to the breakdown of the teeth’s supporting system. Periodontitis is triggered by the accumulation of various microflora (especially anaerobes) in the pockets, which release toxic substances and digestive enzymes and stimulate the immune system. Periodontitis can be efficiently treated using a variety of techniques, both regional and systemic. Effective therapy is dependent on lowering microbial biofilm, minimizing or eradicating pockets. Nowadays, using local drug delivery systems (LDDSs) as an adjuvant therapy to phase I periodontal therapy is an attractive option since it controls drug release, resulting in improved efficacy and lesser adverse reactions. Choosing the right bioactive agent and mode of delivery is the foundation of an efficient periodontal disease management approach. The objective of this paper is to shed light on the issue of successful periodontal regeneration, the drawbacks of currently implemented interventions, and describe the potential of locally delivered repurposed drugs in periodontal tissue regeneration. Because of the multiple etiology of periodontitis, patients must get customized treatment with the primary goal of infection control. Yet, it is not always successful to replace the lost tissues, and it becomes more challenging as the defect gets worse. Pharmaceutical repurposing offers a viable, economical, and safe alternative for non-invasive, and predictable periodontal regeneration. This article clears the way in front of researchers, decision-makers, and pharmaceutical companies to explore the potential, effectiveness, and efficiency of the repurposed pharmaceuticals to generate more economical, effective, and safe topical pharmaceutical preparations for periodontal tissue regeneration.

## 1 Introduction

Periodontitis, a prevalent inflammatory illness, has been designated by healthcare professionals as the third-greatest contributor to mortality in humans, behind only cancer, heart disease, and brain disorders. Its prevalence is between 5% and 25% of the worldwide population, whereas mild periodontitis affects almost 60% (Papapanou and Susin, 2017). Furthermore, the prevalence and frequency of periodontitis are greater among those with limited resources and elderly people. Persistent periodontitis hurts the standard of living and is associated with worry, inadequacy, lack of tolerance, bad odor, and other symptoms. These implications may result in social hurdles and some difficulties with function, including chewing problems.

Mechanical debridement is the primary therapy for periodontitis now. Scaling, and root planning (SRP) physically eliminate harmful microbiota and slow the regrowth of periodontitis-causing bacteria. Nevertheless, due to inadequate visibility into the micro-organisms that reside deeply in the periodontal defect, and the dental intricate structure, SRP cannot entirely eradicate infections (Jhinger et al., 2015).

Furthermore, periodontitis, as an irritation linked with bacteria, can result in alveolar bone loss and destruction of tissues. As a result, SRP can enhance the effectiveness of other medications by combining them with antimicrobial treatments, anti-inflammatory medications, and therapies that stimulate bone and tissue regeneration. The total course of treatment is dependent not only on the medication characteristics but also on the delivery device and routes of delivery (Rams and Slots, 2023). In this setting, the article focuses on the utilization of locally applied repurposed drugs in the management of periodontal illnesses, highlighting current problems and prospective study prospects.

## 2 Pathogenesis of periodontitis

Periodontitis is an inflammation illness with complex causes. In 1997, Page and Kornman devised a traditional framework to explain the complicated etiology (Baru et al., 2024). The traditional theory was constantly changed over the next 20 years to develop the current theory according to novel insights. It is now acknowledged that pathologic dysbiosis is crucial, but not the only reason for the emergence of periodontal diseases. The illness is produced by complicated interactions between dysbiosis and the immunological reaction of the host. There are several fresh perspectives concerning the progression of periodontitis according to modern theory. Obtaining and sustaining therapeutic wellness necessitates an optimal microbiome in which intricate interrelated interplay between the biofilm and the immunological response emerge (Nasiri et al., 2023). If the fragile equilibrium between the dysbiosis and the host reaction is disrupted, a greater host reaction will be generated. Excessive inflammatory cascades are frequently produced, overpowering their opposing competitors and causing periodontal destruction (Trindade et al., 2023). This condition is known as gingivitis because it has not progressed to the periodontitis phase (Meyle and Chapple, 2015). Once bacteria have accumulated to produce dysbiosis, inflammatory cascades take over at the current lesions (Herrmann and Meyle, 2015; Jain et al., 2008). When the chemotactic and bactericidal activities are disrupted, and neutrophils are unable to generate pro-resolving lipid cytokines, the excessive immune reaction causes tissue destruction and adhesion loss, resulting in periodontitis (Sonnenschein and Meyle, 2015). Patient behaviors, drugs, and environmental impacts including lifestyle are examples of changeable variables. All of these variables may give rise to the formation of periodontitis (Petersen and Ogawa, 2005). Periodontitis is a complicated oral disease with various pathogenic variables, including heredity, gender, certain systemic disorders, and epigenetic impacts. Patient behaviors, drugs, and environmental effects like lifestyle are examples of changeable parameters (Yousefi et al., 2020; Liang et al., 2020).

While dental implants possess successful outcomes over time, problems related to poor treatment preparation and implementation, material failure, and infection have been described (Rokaya et al., 2020). The primary biological problem is peri-implantitis, which is defined as an infectious disease triggered by microbiological colonies affecting the tissues enclosing implants, marked by clinical indicators of inflammatory and radiological loss of bones (Belibasakis and Manoil, 2021). A significant proportion rate has been found, and the number of patients impacted could be approximately twenty percent (Diaz et al., 2022). Several therapy approaches have been proposed to treat peri-implantitis. Nevertheless, no advantage of a specific therapy over another has been established, and advanced techniques were unable to show extra advantages over simple therapies (Ramanauskaite et al., 2022).

## 3 Topical drug carriers for management of periodontitis and peri-implantitis

**FIGURE 1:**
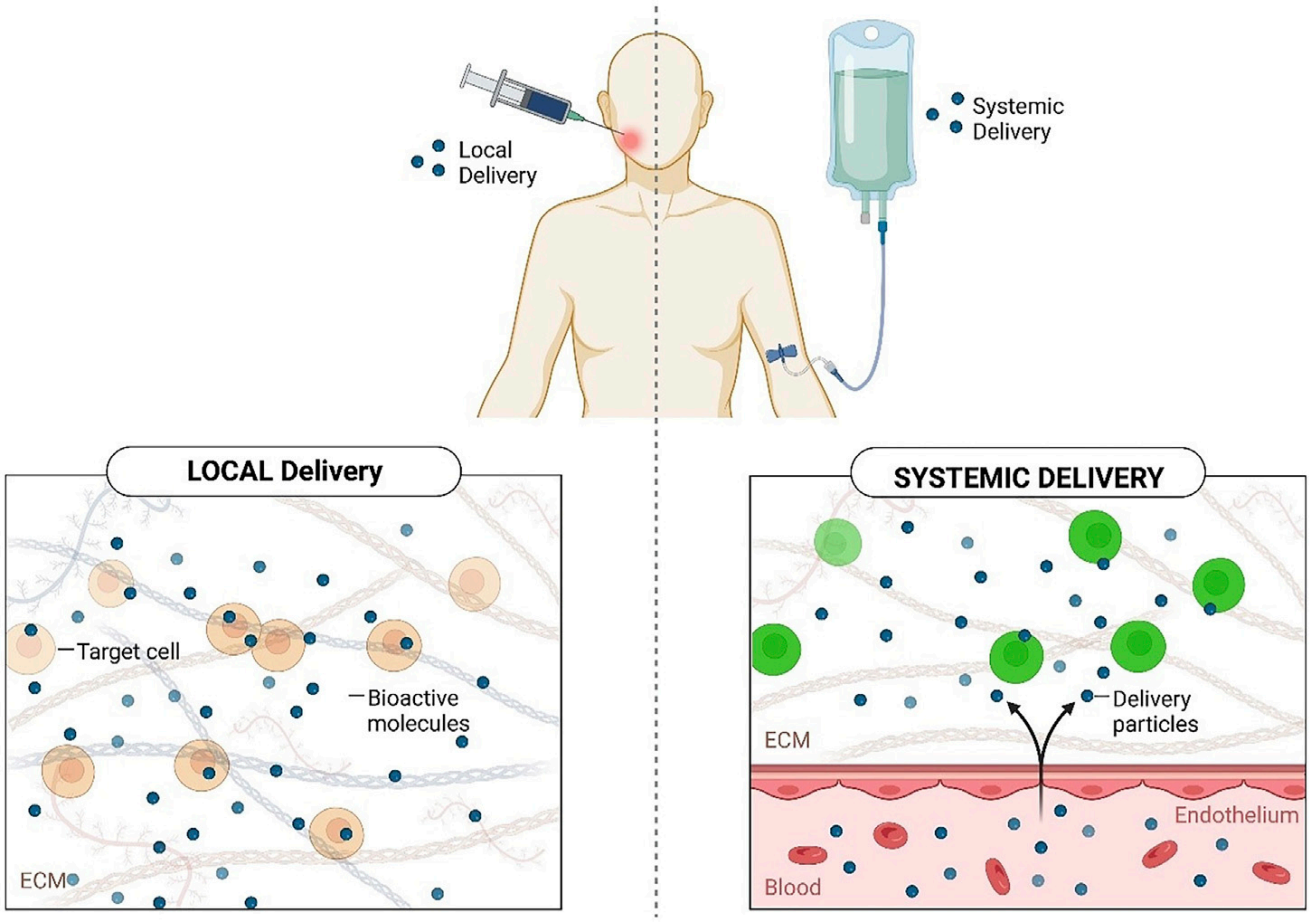
Comparison between local and systemic drug delivery.

Both systemic and topical delivery are essential ways of medication administration. The implementation of a systemic approach in the management of oral dysbiosis has demonstrated some benefits during the last half-century (Rokaya et al., 2020). Nevertheless, systemic medication administration can cause issues that include dysbacteriosis, and poor biodistribution. Furthermore, to acquire and sustain an efficient level, a large dosage is frequently delivered, which could cause toxic effects, digestive discomfort, and resistance to them (Figure 1) (Atia et al., 2024b; Atia et al., 2024a; El-Nablaway et al., 2024a; El-Nablaway et al., 2024b).

Because of the evident difficulties of systemic administration, it is critical to adopt LDDS for improved periodontitis management and avoidance.

Peri-implant disorders, like periodontal illnesses, are mostly caused by tooth plaque. Periodontal health is controlled by a variety of variables, including oral hygiene, genetics and epigenetics, overall health, and diet (Monje et al., 2022). Peri-implantitis and periodontitis lesions contain Gram-negative anaerobic bacteria. Nevertheless, peri-implantitis has a greater greater variety of bacteria than periodontitis (Taymour et al., 2024). Furthermore, peri-implantitis is primarily infiltrated by inflammatory cells, and it commonly doesn't have a protective tissue layer around the bone, which is typical of periodontitis (Sadek et al., 2024). Peri-implantitis lesions were twice as big, exhibited greater blood vessels, and had an infiltration in the connective tissue than periodontitis (Carcuac and Berglundh, 2014). Peri-implantitis had a 97% higher concentration of matrix metalloproteinases (MMP), such as MMP-8, but chronic periodontitis had just a 78% increase compared to healthy gingiva (Zhang et al., 2018). Additionally, peri-implantitis tissue includes extracellular matrix antibodies (Papi et al., 2017). Peri-implantitis progresses more quickly than periodontal disease, resulting in quicker and more extensive bone loss. Peri-implantitis causes a nonlinear kind of continuous bone degradation over time, possibly due to changes in bacteria at the implant locations, the host’s defensive mechanism, and the absence of a periodontal ligament (Hasturk and Kantarci, 2015).

Topical placement of LDDS in the periodontal pocket can produce an adequate quantity of pharmaceutical medicines for a longer duration of time. When juxtaposed with systemic administration, LDDS has additional benefits including avoiding problems within the digestive system and bioavailability by administration at a particular location, greater effectiveness, and fewer adverse reactions by managing the release of medication, and enhanced compliance among patients through decreased periodic dosing. Given these benefits, LDDS has been studied as a method for periodontitis therapy in the past few decades (Viglianisi et al., 2023).

## 4 Drugs integration strategies in drug delivery systems

**FIGURE 2:**
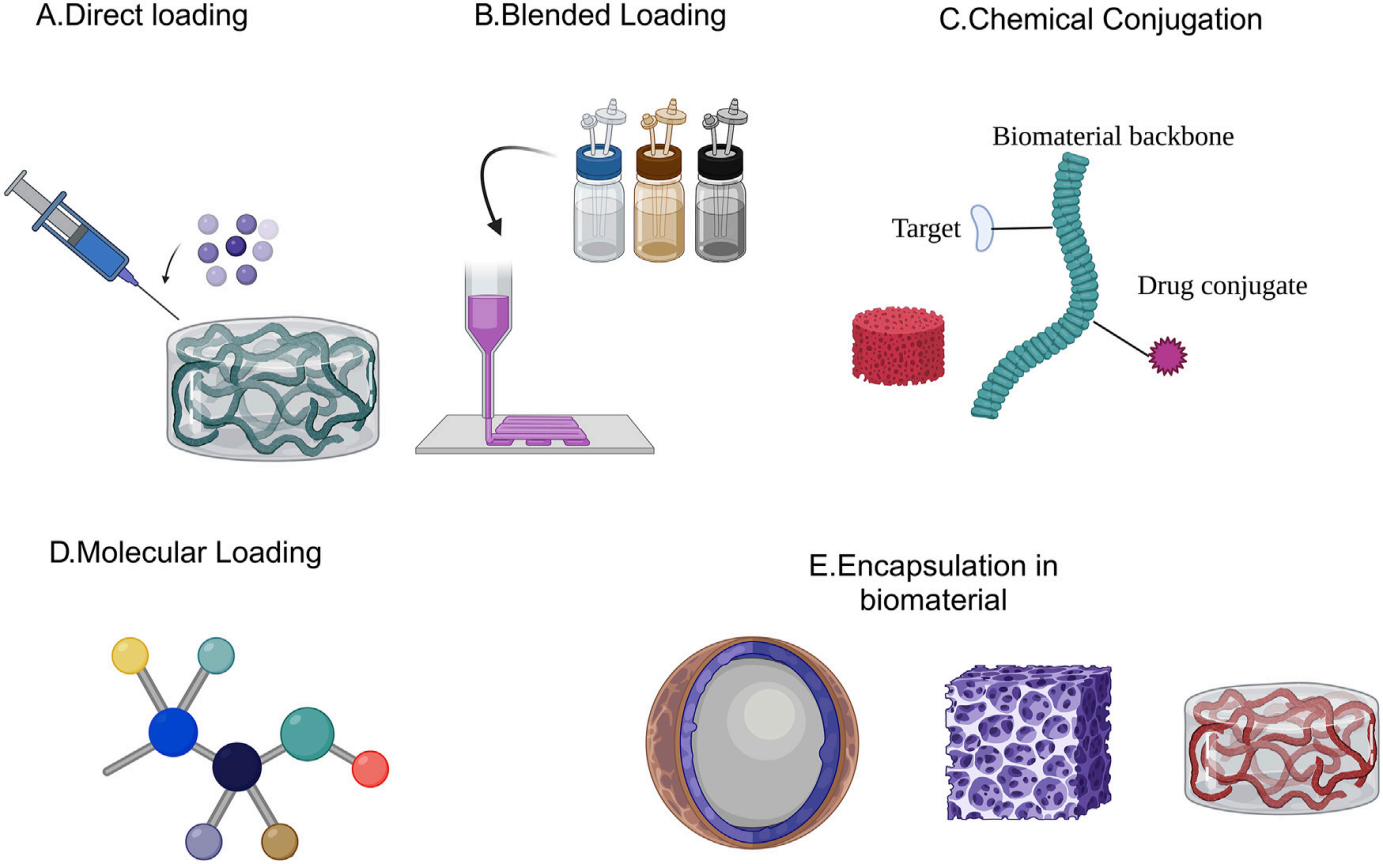
Different techniques for incorporation of pharmaceuticals within drug carriers.

Several approaches, including direct loading, combining, and surface/chemical interaction, have been developed to introduce various medicines into scaffolds for therapeutic purposes (Figure 2) (Tallawi et al., 2015). These strategies have significant benefits and downsides in terms of production procedures, scaffold design and required scaffolding dimensions, customized treatments, controlled drug release, and specific medical applications.

### 4.1 Direct loading

The direct loading method requires submerging the scaffolding in a medicinal solution, which allows chemicals to attach to the framework through physical adsorption and/or absorption. Biomaterials containing bone morphogenic protein-2 (BMP-2), for instance, are a viable method of inducing bone development. DeConde et al. demonstrated the effectiveness of bio active scaffolds treated with BMP-2 in osseous healing in mandibular lesions in rats (Reis, 2019). The downside of this method is that the capacity for carrying is strongly dependent on the scaffolding design, involving the dimensions of the particles, scaffolding unbound volume, moisture absorption, and medicinal physical qualities (e.g., molecular capacity) (Wang et al., 2019).

### 4.2 Blended loading

Unlike direct loading, blending involves integrating medications into the scaffolding framework before constructing the 3D design. This is often performed through the combination of pharmaceuticals and a polymer in a common media that will be used to construct the scaffolding. This method has the advantage of being adaptable to different scaffold biomaterials, pharmaceuticals, and production techniques (Calori et al., 2020). In this regard, electro-spinning has been employed to build nanofiber scaffoldings for nerve regeneration utilizing a combination of vitamin B5 and PLCL/silk composites (Ye et al., 2019). Blending techniques can also be utilized to develop scaffolds with additive manufacturing technologies for purposes that require complex architectures. Additionally, this carrying method has negligible effect on the pharmacokinetic pattern.

### 4.3 Chemical and surface conjugation

Chemical conjugation of biologically active substances to scaffolding elements or surfaces enables the removal of variations between biologically active substances, scaffolding, pharmaceutical loading, and, most importantly, the controlling of breakdown rate for ongoing, induced, or upon request medication discharge (Jiang et al., 2019). Scaffolding surfaces, for example, can be changed to induce purposeful biological reactions that aid in drug binding. In contrast to traditional scaffolding, the development of these bioactive components has been reported to increase the integration of bioactive cues, while also lowering the immediate impact and expanding the discharge length (Freeman et al., 2021).

Chemical conjugation and surface enhancement can help in establishing covalent bonds to scaffolds, allowing medicines to remain on the scaffold constituents or surface. Due to the particles’ remarkable adhesion, the biochemical immobility method is appropriate for postponed and prolonged delivery of medical drugs. It additionally restricts the chemical composition of the pharmaceuticals that can be loaded. Furthermore, unless significant scaffold breakdown allows for effective release characteristics, the immobilizing approach may not be appropriate for drugs that need to be ingested or interact with cell nuclei (Fu and Yang, 2023).

### 4.4 Loading bare biomolecules versus encapsulation/loading

Medicinal compounds can be integrated into scaffoldings in their basic state or entrapped in (or conjugated with) an array of nanocarriers. The latter technique has been utilized to circumvent compatibility concerns between scaffolding and therapies by adding biological components into nanoparticles (NPs) prior to their insertion into frameworks (Xu and Burgess, 2012). Loaded particles can then be introduced into a polymeric matrix before or after scaffolding formation, utilizing the methods outlined above for direct loading, blended loading, and chemical attachment. Multifunctional nanoparticles are another way to put a wide range of medications into frameworks. Yang et al. demonstrated the utilization of painted scaffolds as a carrier for biological molecules, influencing biological activity (Yang et al., 2010).

## 5 Strategies for drug release

**FIGURE 3:**
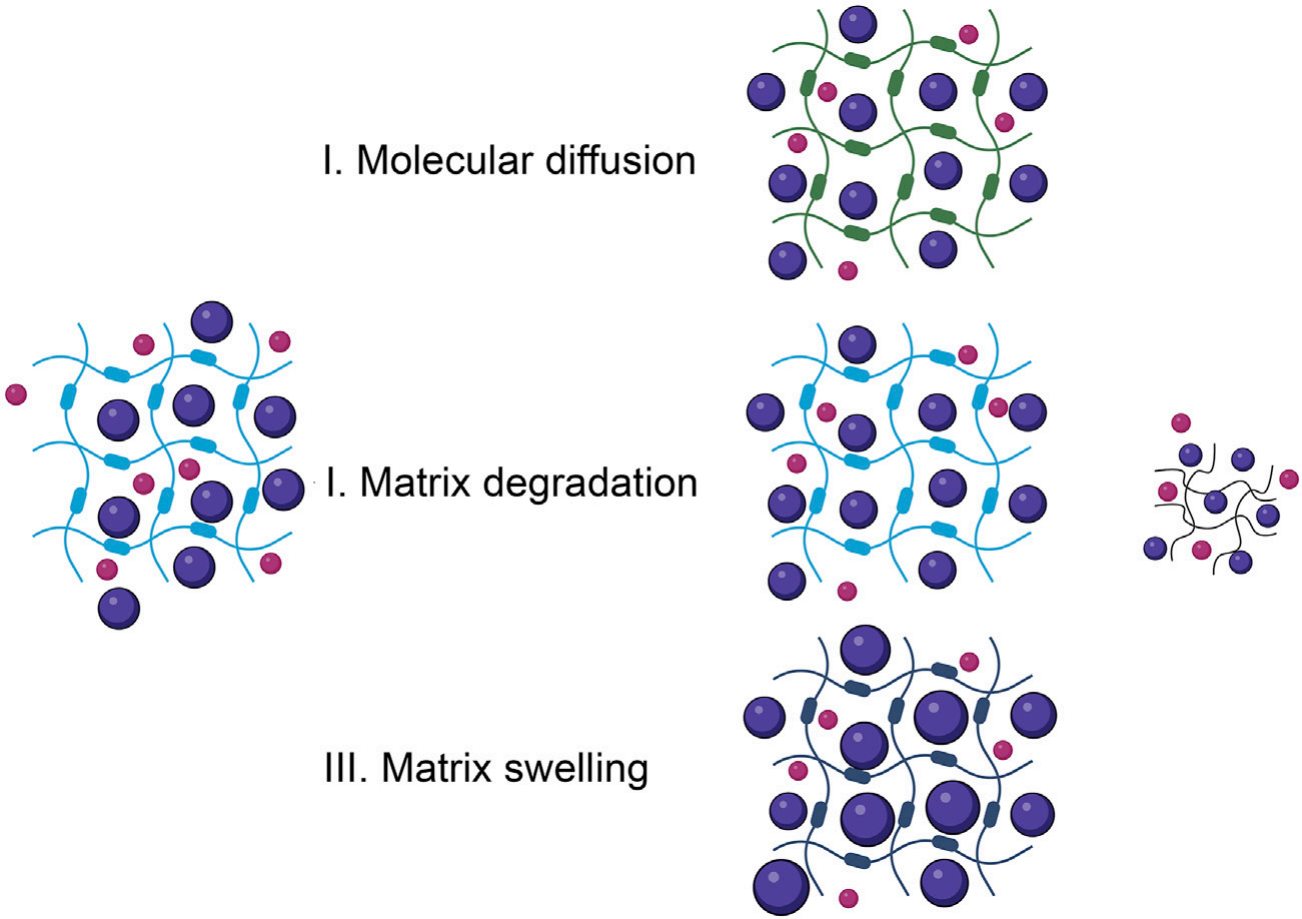
Different mechanisms of drug release.

It is critical to create 3D scaffolds that match the physiological, mechanical, and metabolic functions of ECM. Scaffolds are supposed to execute a number of functions, such as signaling cellular development and activity and transporting biological and medicinal chemicals. The medication delivery approach in porous scaffolding devices can be controlled by either passive or active transportation of bioactive substances in the bioconstructs (Figure 3) (Rocha-García et al., 2016).

### 5.1 Diffusion

The concept of molecular diffusion can be applied to improve medication pharmacokinetics in porous carriers. This phenomenon involves the movement of particles from a large quantities region to a low-volume area. To study the release mechanism in the framework, it is assumed that the pharmaceutical molecules are surrounded by inactive membranes and that the velocity of diffusion is constant (Ridolfo et al., 2020). The volume of the matrix in numerous scaffoldings may affect diffusion speed because a larger free volume allows more water to penetrate the scaffolding, allowing for more drug diffusion and desorption. Mesh dimensions, in addition to open space, are an important factor that might limit the specific dimensions of targeted therapeutic substances (Wanat, 2020). To overcome this drawback, mesh size can be modified by varying the amounts of polymers and cross-linking agents employed in scaffolding assembly.

### 5.2 Burst effect

Another common method of administering drugs is burst release. This signifies the unregulated initial distribution of a large amount of medication. For some therapies, including wound healing, such an impact has been used as an optimal medicine administration technique, where an initial quick release may speed up healing processes and minimize discomfort in patients (Isik et al., 2020). Yet for certain medicinal uses, fast release might be harmful to patients. The quick release has been connected to multiple variables, such as drug-loading situations and the properties of hydrogels and bioactive substances (Mirzaeei et al., 2022).

To mitigate the detrimental consequences of burst release, several strategies have been devised, such as employing cross-linked coatings, increasing the crosslinking proportion in the hydrogel, and developing polymeric blends (Zupancic et al., 2016), Heterogeneous packaging of medicinal components with increased levels in the scaffolding, In molecular submerging, increasing the density of the overlaying layer, as well as constructing a layer-by-layer structure with various medicine and polymer ingredients (Ren et al., 2023).

### 5.3 Affinity-based releasing strategy

The interaction between medications and frameworks can be controlled to alter pharmaceutical pharmacokinetics in scaffolding. The structure, permeability, and structure of the scaffolding have significant effects on the discharge kinetics in passive drug delivery, with little interaction between the drug and the scaffolding. Further biochemical communications can be developed between the scaffolding and the drug to increase control over the release process and kinetics. Chemically, this includes the hydrolysis-mediated degradation of polymeric components, as well as enzyme activity (Abedi et al., 2022). However, particular blends with significant promise for the therapeutic molecule(s) can be incorporated into the polymer results in an extended-release pattern. Cyclodextrins are a well-known macromolecule family that has a strong affinity for numerous therapeutic chemicals (CDs) (Mealy et al., 2015). It exhibited the sustained administration of a drug (L-tryptophan) in a CD-adjusted hyaluronic acid (HA) hydrogel for 21 days. The releasing kinetics showed a prolonged progressive pattern, with only 20% of the medicine delivered following 24 h, compared to 90% in standard HA hydrogel. As an alternative technique, NPs have been integrated as a pharmaceutical transporters, allowing for greater flexibility in tailoring the pharmacokinetic pattern by modifying the NP-polymer interconnections. For instance, after strengthening with silicate NPs, the medication delivery rate of hydrogels was lowered, indicating that NPs limit drug diffusion, decrease swelling, and/or inadequately adhere to the drug. The linkages between the active ingredient and the NP, or the NP and the framework, as a drug transporter, can be changed to control release (Zhang et al., 2022a).

### 5.4 Stimulus-based discharge

#### 5.4.1 Releasing mechanism relying on the scaffolding

In conjunction with passive monitoring, modulation of scaffolding has been developed for different medical purposes by exploiting their sensitivity to external components like acidity and natural stimuli such as glucose (James et al., 2014). For example, a temperature-sensitive polymer scaffolding was used to distribute drugs like aspirin (Du et al., 2018b), erythropoietin (Xu et al., 2019a), and ornidazole (Ravishankar et al., 2017) to treat periodontal disease.

#### 5.4.2 Discharge method dependent on the loaded components

NPs can be used as a signaling molecule to initiate drug release when subjected to initiating conditions such as light. The photothermal heating of AuNPs in a thermally sensitive hydrogel permitted light-controlled drug release. When subjected to near-infrared (NIR) light, carbon nanostructures, and graphenematerials exhibit a photo thermal effect, which has been utilized for light-triggered pharmaceutical dissolution (Trucillo, 2022).

## 6 Drug repurposing

Repurposing a drug entails employing medicines that have been approved by regulatory bodies for a new application. To be licensed for sale, a novel drug must meet stringent standards. Due to the many physicochemical properties of chemical entities, as well as the difficulty of expanding production, finding a drug along with improving it needs substantial investment (Vaidya et al., 2019). This limitation also permits pharmaceutical businesses or educational organizations to utilize already-approved medications quickly and successfully for an unfamiliar purpose to which those suffering from that disease have yet to access (Table 1).

**TABLE 1:** The advantages and the limitations of drug repurposing.

Significance	Obstacles	Reference
Guarantees security	Insufficient knowledge of legal requirements	Jourdan et al. (2020)
It reduces both time and expenses	Inadequate economic motivations	Jourdan et al. (2020)
Marketing potential: rising global revenue spurs growth in markets	The likelihood of failing evidence of concept trials for new purposes is one of the clinical trial concerns	Padhy and Gupta (2011)
Out licensing possibility: novel applications while retaining ownership of the old purpose	Patent restrictions prevent the selling of repurposed molecules	Padhy and Gupta (2011)
Unmet therapeutic requirements can be met by developing novel uses for current pharmaceuticals to treat unusual conditions and attacking tumors with non-cancer therapy	Evaluation of financial requirements	Padhy and Gupta (2011)

All toxicity, experimental, and clinical trial efficacy data for a repositioned medicine are readily available, allowing the researcher to reach an objective decision at every step of pharmaceutical research, and developmental approaches (Pan et al., 2022; Alghonemy et al., 2024; Mohamed, 2024). The accessibility of current knowledge on safety, efficacy, and the right distribution approach significantly reduces investigation expenses and a period leading to less effort required to successfully bring a recycled drug to the marketplace (Abdel Nasser Atia et al., 2022).

Because of the tremendous potential of a shorter development process, several drug companies are currently utilizing medication repurposing to reassemble authorized, as well as previously unsuccessful substances, into fresh medications for a range of medical conditions (Badria, 2020).

## 7 Repurposed drugs for local periodontal regeneration

**FIGURE 4:**
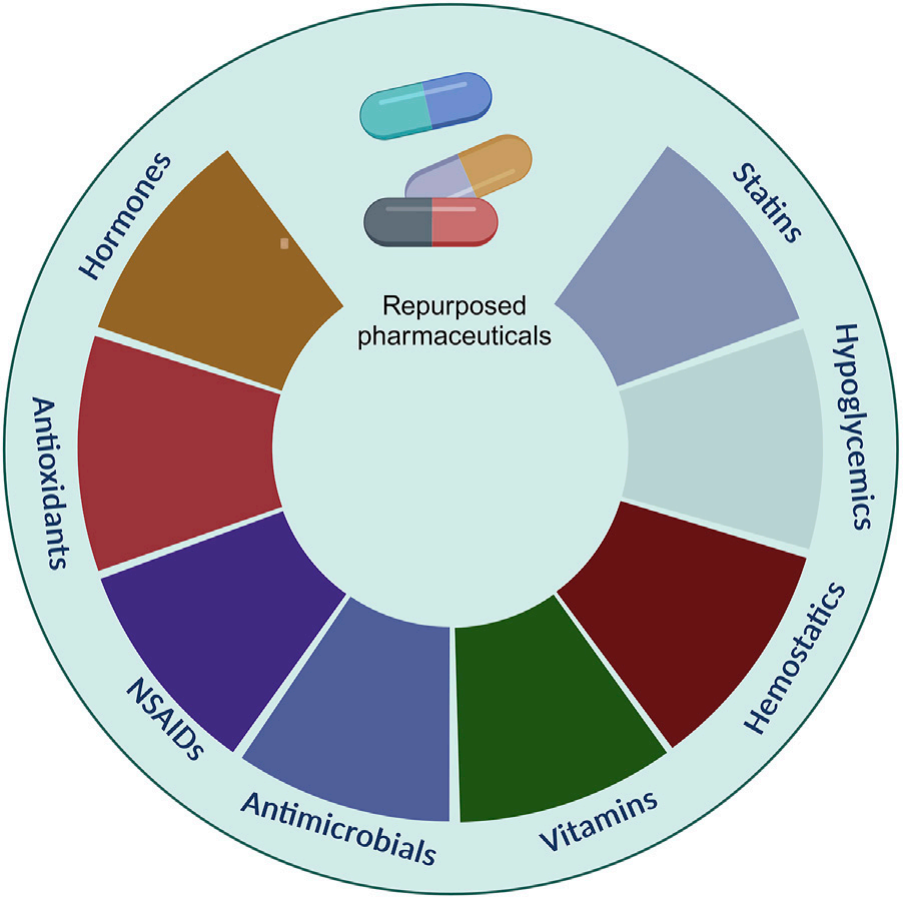
Examples of repurposed pharmaceuticals for periodontal regeneration.

There is a plethora of pharmaceuticals that could be repurposed for implementation in periodontal regeneration (Figure 4).

### 7.1 Nonsteroidal anti-inflammatory drugs (NSAIDs)

They are medications that are widely employed to manage pain, decrease inflammatory conditions, and lower fever (Díaz-González and Sánchez-Madrid, 2015). NSAIDs showed promise in periodontal regeneration (Table 2) (Preshaw, 2018).

**TABLE 2:** Application of locally delivered repurposed NSAIDs for periodontal regeneration.

NSAIDs	Carrier	Model	Outcome	Reference
Aspirin	CS/ β -GP/G hydrogel	Rats	Excellent cytocompatibility. Anti-inflammation and periodontal regeneration effects	Xu et al. (2019b)
	hydroxyapatite/tricalcium phosphate (HA/TCP)	Mice	Enhanced osteogenesis	Wu et al. (2019)
	Platelet-rich fibrin	Rats	Prolonged discharge of aspirin/salicylic acid. Encouraged periodontal ligament mesenchymal cell growth and movement	Du et al. (2018a)
		Human PDLSCs	ASA increased the activity of markers linked to cellular growth, and osseous regeneration	Abd Rahman et al. (2016)
	Tetra-PEG hydrogel	PDLSCs Mice calvaria bone defects	Increased PDLSCS multiplication, and osteogenic differentiation	Zhang et al. (2019)
Meloxicam	CS/PVA/HA electro-spun fibers and films	Epithelial cells of African green monkey (VERO)	Sustained release of meloxicam	Yar et al. (2016a)
	3D chitosan Scaffold	L929 mouse fibroblasts	The scaffold had a porous structure Cytocompatibility *in vitro* Meloxicam is released continuously	Nowak et al. (2019)
Ibuprofen	PCL/PVA/COL Nanofiber	*In vitro*	Outstanding cytocompatibility Sustained drug release	Limoee et al. (2019)
	PLGA/CHX chlorohexidine	Human oral epithelial cells P. gingivalis strain. male C57BL/6J mice	TNF-release suppression Bactericidal activity	Batool et al. (2019)
	Calcium-deficient hydroxyapatite/tricalcium phosphate (β-TCP)	*Escherichia coli* Albumin denaturation method Swiss 3T3 fibroblast cells Rat cranial defects	Outstanding biocompatibility Anti-inflammation, and antimicrobial activity	Madhumathi et al. (2018)
Piroxicam	CS/PVA/HA nanofibers	*In vitro*	Prolonged release of piroxicam, and optimal biomechanical properties	Farooq et al. (2015)

CS, chitosan; β-GP; Beta-glycerophosphate, G, gelatin; EPO; erythropoietin; RLX, raloxifene; TCP, tricalcium phosphate; PCL; polycaprolactone, PVA; polyvinyl alcohol, TNF; tumor necrosis factor alpha, CHX; chlorohexidine, PEG; polyethylene glycol, PDLSCs, Periodontal ligament stem cells; Col, Collagen; Cs; Chitosan; HA, hydroxy apatite.

#### 7.1.1 Acetylsalicylic acid (ASA)

It is additionally referred to as aspirin and can accelerate bone healing. Numerous publications have been written about its physiological functions, the vast majority of which are linked to other immune system regulation, including T-cell inhibition, MSC longevity prolongation, and immune regulation ability enhancement (Liu et al., 2011). ASA-based chitosan hydrogels showed long-term dispersal for more than 14 days, enhancing PDLSC replication and bone formation (Zhang et al., 2022b). CS/b-GP/G/EPO/RLX hydrogels are effective for treating periodontal disease (Xu et al., 2019b).

#### 7.1.2 Ibuprofen (IBU)

Ibuprofen has anti-inflammation characteristics similar to aspirin but causes considerably less digestive pain. Thermo-sensitive microgels carrying metronidazole (MET) and ibuprofen (IBU) displayed excellent MET discharge for 8 h and sustained IBU release for up to 48 h, demonstrating that they could be used in periodontal regeneration (Pham et al., 2021).

#### 7.1.3 Meloxicam

Meloxicam helps to keep cartilage and bone in place. Noncytotoxic electro-spun (e-spun) chitosan (CS)/poly vinyl alcohol (PVA)/hydroxyapatite (HA) fibers and films filled with meloxicam were discovered, encouraging cellular division. These features, together with these compounds’ immune system regulation capacity, hint at possible utility in periodontal therapy (Yar et al., 2016b).

#### 7.1.4 Piroxicam

CS/PVA/HA nanofibers loaded with PX resulted in prolonged release of PX, and optimal biomechanical properties (Farooq et al., 2015).

### 7.2 Statins

Statins are potent drugs for cholesterol reduction that work by inhibiting a critical component of the cholesterol manufacturing process. They have made significant advances in the avoidance of cardiac events. Statins have anti-inflammatory and immune-modulating capabilities since they suppress the generation of inflammatory cytokines like interleukin 1 (IL-1) and interleukin 6 (IL-6), as well as tumor necrosis factor (TNF) (Koushki et al., 2021). Since Mundy et al. initially explored statins’ bone anabolic effects nearly 15 years ago, there has been a vigorous hunt for potential applications to bone catabolic illnesses like osteoporosis (Mundy et al., 1999).

Statins can also promote osteoblast formation by increasing bone morphogenetic protein-2 (BMP-2) levels, which are thought to be an inducer for osteoblast transformation and bone creation (Dalcico et al., 2013). They may additionally boost bone tissue formation by activating the vascular endothelial growth factor (VEGF) (Balli et al., 2014). Statins, because of their anti-inflammatory and osteo-inductive capacities, have been proven to be helpful in the enhancement of osteogenesis in human periodontal ligament cells (Table 3) (Mundy et al., 1999). Statins enhance osteogenesis by promoting the division and transformation of osteoblasts, while also protecting these cells from apoptosis. Additionally, statins reduce osteoclastogenesis by inhibiting the differentiation of osteoclasts (Pacheco-Pantoja and Alvarez-Nemegyei, 2014; Moshiri et al., 2015).

**TABLE 3:** Local applications of statins in periodontal regeneration.

Statin	Scaffold	Model	Outcome	Reference
Atorvastatin	thermosensitive chitosan hydrogel	*In vitro* and *In vivo*	BSP2 upregulation in osteoblasts highlights atorvastatin’s healing properties	Pradeep et al. (2016a)
	Methylcellulose gel	Diabetic humans	Enhanced periodontal regeneration, and bone fill	Kumari et al. (2016)
Lovastatin	1 Cs/epigallocatechin-3-gallate membrane	Dogs	Lovastatin long-term distribution aided osteogenesis. EGCG14-CS had promise antibacterial action; and promoted periodontal repair	Lee et al. (2016a)
	2 Polyurethane (PUR)	Rats	The quantity of NFB was much higher in lovastatin-PUR specimens	Yoshii et al. (2010)
Fluvastatin	PGA	Rabbits	Enhanced osteogenesis, and osseointegration	Moriyama et al. (2008)
	Gelatin hydrogel	Rats	Significant osteoinduction	Tanabe et al. (2012)
	Collagen graft	Rabbits	Simvastatin-collagen scaffolds induced osteogenesis	Wong and Rabie (2003)
	Gelatin hydrogel	Rats	Enhanced angiogenesis, and osteogenesis	Fukui et al. (2012)

CS, chitosan; PUR, polyurethane; PGA, poly glycolic acid.

#### 7.2.1 Mechanism of actions of statins

**FIGURE 5:**
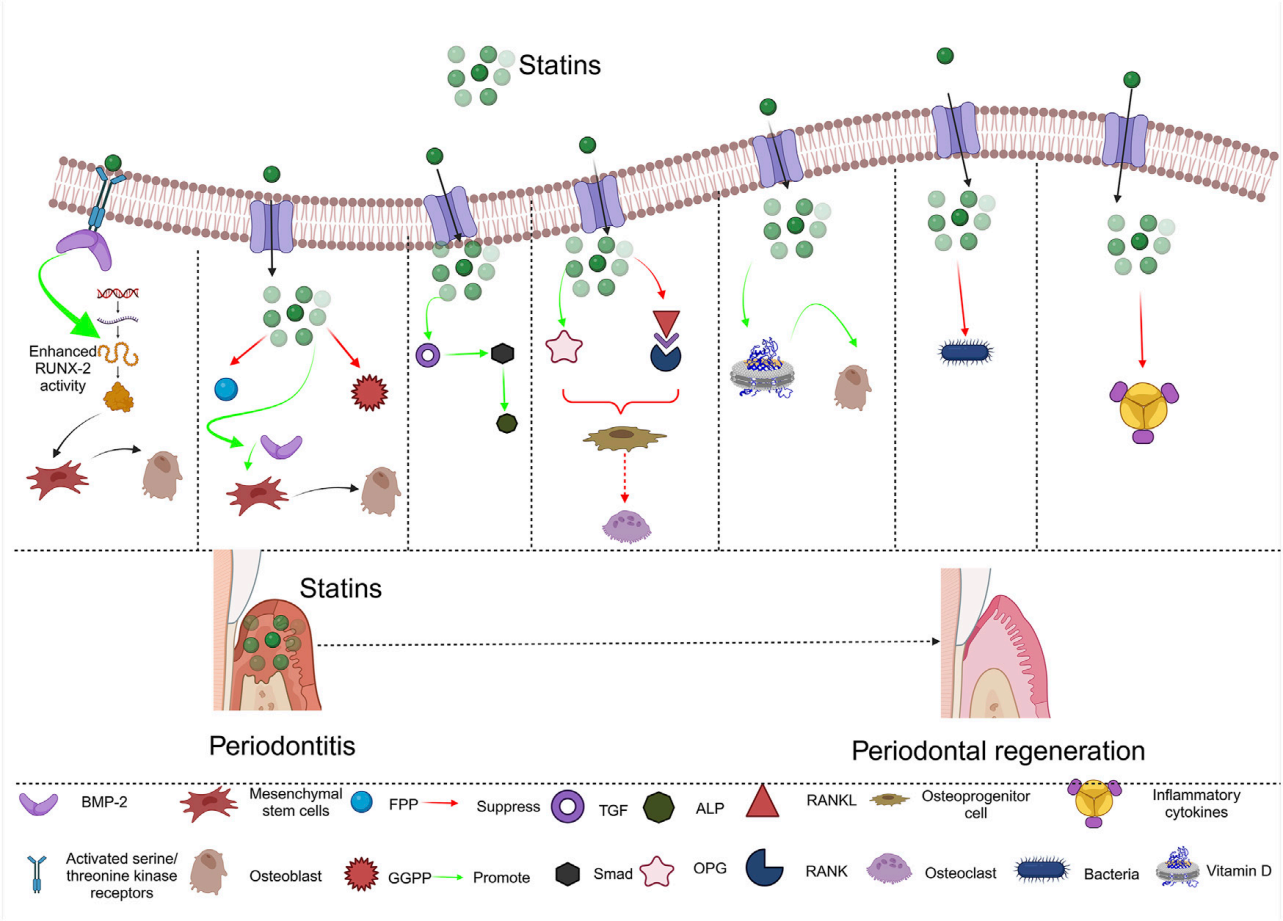
Mechanism of action of statins in periodontal regeneration.

Statins have periodontal regeneration-promoting characteristics, such as being anti-inflammatory (Satny et al., 2021), promoting the growth of bones (Figure 5), inhibiting tissue metabolizing enzymatic processes, and possessing antimicrobial capabilities (Hennessy et al., 2016; De Giorgi et al., 2023).

##### 7.2.1.1 Statins promote osteoblast development by increasing BMP-2

Statins stimulate new bone formation (NBF) by increasing BMP-2 transcription. Lipophilic statins, such as simvastatin can stimulate the BMP-2 gene regulator; whereas more hydrophilic statins, including pravastatin, do not (Wan Hasan et al., 2020; Li et al., 2023). At the cell membrane, BMP-2 interacts with a particular type II receptor, forming a complex by engaging a type I receptor. The compound of BMP-receptor II and BMP-receptor I (activated serine/threonine kinase receptors) Smad proteins are phosphorylated (Wu et al., 2021). Statins may boost Runx2 gene activity via increasing Runx2 expression, which leads to the differentiation of MSCs into osteoblastic cells (Hong et al., 2020).

##### 7.2.1.2 Statins promote osteoblast development and bone matrix mineralization by decreasing the synthesis of FPP and GGPP

Statins modulate downstream byproducts of mevalonate (for example, FPP and GGPP). FFP diminishes during statin-stimulated osteoblast development. Exogenous FPP expression prevents osteoblastic cell differentiation. Lovastatin, for example, lowered the amounts of both FPP and GGPP in cultured cells in a dose-related way (Nes, 2011). GGPP has also been linked to statin-initiated bone formation. GGPP mRNA and protein levels have been demonstrated to decrease during mineralization in MC3T3-E1 cells. Geranyl-geraniol (GGOH), which is transformed to GGPP in the cells, suppresses the differentiation of MC3T3-E1 cells, indicating that GGPP may have an adverse effect on osteogenesis (Weivoda and Hohl, 2011). Pitavastatin-treated osteoblasts expressed more BMP-2 mRNA *in vitro*, but this impact was reversed when subjected to mevalonate or GGPP (Tai et al., 2013). Simvastatin also raised BMP-2 mRNA expression and accelerated MC3T3-E1 cell transformation into osteoblastic cells; nevertheless, cells primed with GGPP inhibited simvastatin-triggered differentiation (Zhong et al., 2011).

##### 7.2.1.3 Statins suppress osteoblastic apoptosis via the TGF/Smad3 signaling system

Statins protect osteoblasts from apoptotic processes through the TGF/Smad3 signaling pathway. TGF stimulates Smad3 by launching several actions that result in the stimulation of a specific enzyme (Neve et al., 2011). By producing matrix proteins and enhancing ALP functionalization and mineralization, Smad3 promotes osteogenesis. Statins increase Smad3 expression in osteoblasts, which may help to prevent dexamethasone-induced apoptosis.

##### 7.2.1.4 Statins suppress osteoclastogenesis by increasing er transcription via the OPG/RANKL/RANK signaling cascade

Statins influence osteoclast regeneration by modulating the OPG/RANKL/RANK signaling pathway. There is a link between statins and estrogen (ER) (Song et al., 2008). It has been demonstrated that countering the BMP-2 monoclonal antibody did not completely inhibit ALP activity, whereas estrogen receptor-alpha (ER-α) protein concentrations boosted following dose-dependent simvastatin treatment of mouse BMSCs, implying that simvastatin preserves BMD by inducing either ER or BMP-2. Simvastatin enhanced Er expression in the bone in a synergistic way with estrogen in ovariectomized rats (Song et al., 2008; Nyan et al., 2007; Chen et al., 2010; Albu et al., 2013). They could boost OPG mRNA concentrations while decreasing RANKL gene expression in mouse bone cells (Chamani et al., 2021).

##### 7.2.1.5 Statin-vitamin D interactions

Two clinical investigations found that rosuvastatin medication boosted 25-hydroxyvitamin D levels considerably (Yavuz et al., 2009; Ertugrul et al., 2011). This might explain why, in some clinical investigations, the boost in bone mineral density with statin therapy was mediated by an elevation in vitamin D concentrations. Nevertheless, fluvastatin (Ertugrul et al., 2011), and simvastatin (Rejnmark et al., 2010) administration has no effect on the concentrations of 25-hydroxyvitamin D. The process underpinning statins’ impact on vitamin D has not been determined, however, the shared catabolic route for statins and vitamin D may be to blame (Ertugrul et al., 2011). CYP enzymes may potentially affect the influence of vitamin D on statin actions. As a CYP enzyme inducer, vitamin D may boost the metabolism of statins (Bhattacharyya et al., 2012). Active CYP metabolites, such as those seen in atorvastatin, may potentially aid in therapeutic success.

##### 7.2.1.6 Antimicrobial activity of statins

About 700 species colonize the mouth cavity. Although some are regarded as commensals, persistent biofilm formation is the cause of caries, gingivitis, and other dental problems. Statins have antibacterial action, and some studies have found statins to be effective against oral microbes, especially those found in the subgingival colonies, even at reduced levels (Kamińska et al., 2019).

Kaminska et al. revealed in 2019 that statins had antibacterial activities, mostly lowering their production, which may imply a modification in microbial activities. The existing biofilm, on the other hand, was less impacted, with enhanced bacterial viability, despite a drop in *P. gingivalis* and *T. forshythia*. As pathogens declined, the abundance of *S. gordonii* increased, resulting in a shift in populations between harmful and nonpathogenic bacteria. As a result, these results suggest that statins have a significant role in managing the oral biofilm, lowering bacterial load in favor of a greater physiologic microbiome (Kamińska et al., 2019).

##### 7.2.1.7 Impact of statins on immunological attributed inflammation

The formation of dysbiosis biofilm can trigger an increased host response, resulting in the loss of periodontal tissues. If no treatment intervention is implemented, pathogenic bacteria and the imbalanced immune-inflammation-mediated reactions feed each other, resulting in a self-perpetuating loop. When the inflammatory process advances to the alveolar bone in response to a pathogenic stimulation, it stimulates the initiation of bone resorption. The adaptive immune system additionally serves a crucial function in host tissue defense, but, when left unchecked, it may lead to periodontal deterioration.

Statins influence T cell development and antigen presentation in the adaptive immune system. *In vitro*, atorvastatin and simvastatin increased the number of human regulatory T cells (Tregs) and induced CD4 differentiation into Tregs (Petit et al., 2019; Mausner-Fainberg et al., 2008; Kagami et al., 2009). Statins have varying impacts on the release of inflammation-associated mediators. Simvastatin and rosuvastatin reduce IL-6 and IL-8 levels. Simvastatin, on the other hand, enhanced the expression of IL-4, IL-5, and IL-13 in T cells.

They have been proven in animal experiments to be able to suppress the generation of inflammatory mediators in the periodontal pocket of rats, correlating with specific findings observed in *in vitro* research (de Araújo Júnior et al., 2013; Jin et al., 2014b; Jin et al., 2014a; Messora et al., 2017). That’s why they are widely implemented in periodontal therapy approaches for their pleiotropic beneficial effects, on both of prevention of inflammation, and the promotion of regeneration.

#### 7.2.2 Atorvastatin

Atorvastatin is a statin medication intended to decrease the amount of cholesterol in the blood (McIver and Siddique, 2023). In the past few years, the different features of atorvastatin have been investigated for their possible use in the treatment of a variety of inflammatory and immune-related diseases, including periodontitis.

There have been several investigations on the topical delivery of atorvastatin for periodontal treatment, notably with gel devices (Pradeep et al., 2016b; Elavarasu et al., 2012). Nonetheless, atorvastatin has a limited bioavailability due to its weak water solubility. Atorvastatin chitosan gel could counteract inflammatory cytokines and promote bone regeneration (Işılay Özdoğan et al., 2018).

#### 7.2.3 Lovastatin

Lovastatin is employed for the treatment of coronary heart disease, high cholesterol levels, and congenital excessive cholesterol levels in adolescents. Chitosan membranes containing epigallocatechin-3-gallate and lovastatin demonstrated high alkaline phosphatase functionality as well as antibacterial action against prevalent infectious microbes, leading to improved bone repair (Lee et al., 2016b).

### 7.3 Hemostatic agents

ε-aminocaproic acid (εACA) is a plasmin-plasminogen antagonist that is synthesized. Fibrin- εACA–Cs could increase cementogenesis and osteogenesis by modifying fibrin biodegradability, hinting that they could be used therapeutically to promote periodontal regeneration (Park et al., 2017).

### 7.4 Hypoglycemic drugs

#### 7.4.1 Metformin

Metformin is the first-line therapy choice for hyperglycemia in type 2 diabetes. It stimulates the AMPK process, which is a critical energy sensor that induces autophagy and controls metabolic processes in cells to preserve energy balance. To improve the survival of cells, autophagy efficiently inhibits the apoptosis-activated protein caspase-8 (Jiang et al., 2021). The autophagic pathway has been shown to moderate excessive cellular ROS-induced impairment to sustain cellular homeostasis and safeguard the survival of cells during stress. Metformin has been shown to improve osteogenic differentiation and mineralization *in vitro* and increase bone density *in vivo*, in addition to its protective impact (Zhang et al., 2021; Zhang et al., 2024; Ha et al., 2024). Furthermore, it is utilized in conjunction with phase I therapy to address periodontal abnormalities, with considerable repair results (Pradeep et al., 2013; Akram et al., 2018).

##### 7.4.1.1 Mechanism of action of metformin

Following metformin application, the primary known antidiabetic mode of activity is mitochondrial respiratory chain (complex I) inhibition, resulting in oxidative phosphorylation separation and enhanced AMP/ATP ratio. Therefore, a boost in the AMP/ATP ratio activates 5′ adenosine monophosphate-activated protein kinase (AMPK), and AMPK is capable of controlling many additional enzymes (Shafiei-Irannejad et al., 2017). Insulin activates insulin receptor substrate 1 (IRS1) via activation of the insulin receptor (IR), resulting in amplification of the glucose transporter (GLUT) in the cell membrane and enhanced glucose absorption (Chopra et al., 2012). AMPK enhances insulin sensitivity by allosterically activating IR and IRS1 (O'Neill, 2013). Though AMPK improves nutrition intake by increasing responsiveness to insulin, it does not operate as completely as insulin and suppresses anabolic reactions (Friedrichsen et al., 2013). Surprisingly AMPK shifts the body’s metabolism into a catabolic state, creating energy and ATP to maintain normal cell function while increasing insulin sensitivity, glucose, and lipid metabolism, and decreasing gluconeogenesis, particularly in the liver.

Recent research has demonstrated that metformin is potentially bone-promoting *in vitro* after activating AMPK, leading to osteoblastic transformation, bone matrix production, and osteoblast proliferation. According to one investigation, AMPK may induce osteogenesis in MC3T3-E1 cells and decrease adipogenesis in 3T3-L1 cells via the AMPKGfi1-OPN axis (Wang et al., 2016). Another research found that AMPK can inhibit RANKL-induced osteoclast development (Lee et al., 2010). In the setting of two mediators, M-CSF and RANKL, osteoclasts are formed by multinucleated big cells (monocyte-macrophage lineages) (Sun et al., 2021).

Metformin also lowers hepatic lipids by inhibiting lipogenesis and improving lipid metabolism. Metformin, in general, can regulate cell development, division, and death via a variety of signaling mechanisms (Shafiei-Irannejad et al., 2017).

### 7.5 Relocated hormones for periodontal tissue engineering and bone regeneration

Growth factors’ oriented treatments are expensive and can induce adverse events and immunologic responses in some people. To compensate for these drawbacks, different hormones have been developed and evaluated as effective substitutes for growth factors. They are cheap to generate, easy to engineer and create, and have little immune response because of their adaptability (Table 4) (Visser et al., 2016).

**TABLE 4:** Hormones utilized locally for bone and periodontal tissue engineering.

Hormone	Original application	Repositioned implementation	Reference
Thyroxin	Thyroid carcinoma and dysfunction	Revascularization and new blood vessel formation	Malik et al. (2020)
Oxytocin	Bleeding after childbirth, labor stimulation, and imperfect, or unavoidable abortion	Increased bone formation	Park et al. (2014)
Dexamethasone	Rheumatism, hematologic/hormonal problems, allergies, and skin diseases, Visual issues, breathing issues, digestive problems, tumors, and allergic responses	Enhanced bone marrow activity and bone formation enhancement	Zhou et al. (2020)
Androgens	Estrogen generation, sexual desire, and bodybuilding	Improved implant Osseo-integration and bony deformity correction	van de Ven et al., 2021b
Parathyroid Hormone	Calcium/Phosphorus homeostasis	Enhances ALP activity and mineralization	Ning et al. (2019)
Insulin	Management of type I diabetes	Accelerated bone healing, and regeneration	Wang et al. (2018b)
Selective Estrogen Receptor Modulators	Postmenopausal osteoporosis the management and avoidance	Cellular growth, calcification capabilities, and function of ALP have all improved	Mu et al. (2018)
Erythropoietin	Management of severe anemia	Anti-inflammation and periodontal therapeutic characteristics	Xu et al. (2019b)
Estrogen	Ovaries dysfunction Hypogonadism in women	Markers of cellular proliferation and the development of osteoblast were improved	Wang et al. (2018a)
Melatonin	Management of sleep deprivation	Bone formation, and development in preosteoblast cellular lineages	Huang et al. (2020c)
Calcitonin	Management of increased calcium levels, and Paget`s disease	Lowers osseous destruction via regulating osteoclastic activity	Wada-Mihara et al. (2018)
Adiponectin	Regulation of blood glucose management and lipid oxidation	-Reduces inflammation - Possesses anti-apoptotic properties -Promotes the division of osteoprogenitor cells and osteoblasts. Promotes the osteoblastic transformation of HPDL cells.	Fang and Judd (2018), Iwayama et al. (2012)
Angiotensin	Management of volume and blood pressure	-Decreases inflammation -Promotes proliferation of periodontal cells -Enhanced bone regeneration	Singh and Karnik (2016)
Cortisol	Natural hormone released in response to stress, and fear	-Decreases inflammation - Promotes bone regeneration	Botelho et al. (2018), Warren et al. (2014)

Human periodontal ligament; HPDL.

Numerous hormones contribute to the growth, development, and preservation of both periodontium and bone. In general, hormonal influences on periodontal health, bone growth, and maximum bone mass are significant (Stutz et al., 2020; Ortiz-Sánchez et al., 2021). Hormones are molecules produced by the body’s hormone-producing glands and discharged into the circulatory system, where they are delivered to certain target cells to exert the effects they have. Hormones can function as transmitters or as complicated organizers of several vital activities that involve blood volume and pressure controlling, advancement, and reproduction (Fraser et al., 2022; Swanson et al., 2022). The subsequent hormones were discovered to be associated with periodontal regeneration.

#### 7.5.1 Thyroxin

Thyroxin is a crucial molecule that serves several biological tasks in our bodies (Hulbert, 2000). One of these is its ability to promote vascular growth through a multitude of techniques (Aleem et al., 2017). It triggers the synthesis of angiogenesis facilitators by activating integrin v3 (Murk et al., 2013). Thyroid hormones have an impact on cell metabolism in addition to cellular multiplication (Sirakov et al., 2013). Chitosan composites encased in different amounts of thyroxin were shown to be cytocompatible, and these pro-angiogenic hydrogels offer a wide range of potential uses in periodontal regeneration (Malik et al., 2020).

#### 7.5.2 Adiponectin (AN)

It is a hormone generated by fat cells and is commonly associated with blood glucose management and lipid oxidation. Nevertheless, it serves a variety of other physiological needs (Nguyen, 2020). AN is now believed to have the ability to act as a beneficial bone density regulator, angiogenic booster, and osteoclast inhibitor (China et al., 2018; Huang et al., 2021). AN reacts with cementoblasts (OCCM-30), influencing cell movement, division, and cementogenesis partially via the Mitogen-activated protein kinase (MAPK) signaling system (Yong et al., 2020). P38, ERK1/2, and JNK suppression resulted in the stimulation of AN-mediated movement and development to variable degrees, whereas MAPK suppression resulted in enhanced mineralization to different extents (Rodríguez-Carballo et al., 2016; Yong et al., 2021). All of this promotes cell growth and cementogenesis. AN delivery in rabbits led to higher mineral levels, durability, and harder bone structure regionally, implying fresh and quicker bone production (Jiang et al., 2011). The rabbits in the research experienced distraction osteogenesis, a surgical approach of expanding bone by slicing it and progressively tugging the fragments separated over time with a mechanical device. The fundamental processes of AN-stimulated bone repair in surgically created gaps were determined to be the attraction and clonal proliferation of cells responsible for creating bones under mechanical stimuli (Yang et al., 2019).

Furthermore, two AN-receptors were recently discovered as being activated by osteoblasts, suggesting that AN has primary roles in bone metabolic processes, such as encouraging division and increasing osteogenesis (China et al., 2018). Because bone formation depends on proper blood flow, vasculature is an essential part of bone development and functionality. The AN has been shown to impact physiological reactions in endothelial cells in an ischemia environment to enhance angiogenesis (Adya et al., 2015). Ouchi and colleagues found that treatment of AN promoted angiogenesis in mouse and rabbit studies (Ouchi et al., 2004). AN has been shown to treat periodontitis because of its anti-inflammatory and bone-healing properties (Wang et al., 2021). APN has the ability to promote bone formation by upregulating the enamel matrix derivative-induced production of development and osteoinduction-related proteins (Nokhbehsaim et al., 2014).

#### 7.5.3 Oxytocin (OT)

OT is a key anabolic hormone present in mammals during nursing that has both regional and systemic effects on bone remodeling (Colaianni et al., 2012). Bone cells contain OT receptors, and OT is implicated in the mechanism of bone remodeling, as it has been shown to inhibit bone loss while increasing bone creation (Breuil et al., 2021). Furthermore, OT increases osteoblastic development and function, resulting in greater bone production and improved bone structure (Feixiang et al., 2023). Decreased oxytocin levels in the blood have been linked to osteoporosis after menopause.110,111 and have been discovered to be involved in bone equilibrium (Breuil et al., 2011). Therapy with OT has been demonstrated to raise calcium levels within cells and to control the activation of osteoblast production and consequently the generation of bones in rats. Furthermore, in mice, ablation of the OT receptor led to the formation of OP. According to an investigation conducted by Jee et al., OT increases bone loss and results in a favorable bone metabolism throughout alveolar bone repair in female rats (Jee and Ma, 1997). It promotes bone formation by increasing osteoblast growth, osteoclast activity, and BMP2 expression.

Despite being researched for a variety of uses in medicine, the influence of oxytocin on local bone formation has not been addressed, most likely due to its brief duration of action and vulnerability to dissolution (Wang et al., 2024). A polymer hydrogel scaffolding containing spherical oxytocin hormone and biphasic calcium phosphates enhances calvarial bone healing in rats (Akay et al., 2020). Additionally, OT-encapsulated β-TCP promotes osteogenesis in rats with calvaria bone abnormalities through an osteoinduction mode of action (Park et al., 2014). It promoted osteogenic growth, division, and aggregation of PDLSC *in vitro*. In addition, the effect of OT on osteogenic progression was linked to the ERK and AKT processes. As a result, OT could be beneficial in restoring periodontal tissues (Ge et al., 2019).

#### 7.5.4 Dexamethasone (DEX)

DEX has been shown to improve osteoblast growth and osseous formation by increasing genes linked to osteoblasts (Chen et al., 2018; Jørgensen et al., 2004). DEX has long been used as an osteoinductive agent because of its high consistency and osteogenesis (Martins et al., 2010; Li et al., 2015). Excessive DEX amounts, on the other hand, could impede osteoblastogenesis and have potentially adverse reactions (Arafa et al., 2023). Consequently, it has limited further functional utility in bone tissue engineering. Thus, sustained DEX discharge is necessitated to maximize efficiency, meanwhile minimizing detrimental impact on osseous healing. Injectable dexamethasone-loaded hydrogels show promise as an injectable drug repository for bone regeneration treatment in situations of persistent inflammatory reaction (Chauhan et al., 2021).

#### 7.5.5 Angiotensin

Angiotensin is generated in the liver and discharged in an inert condition, where it is divided by the enzyme renin and transformed to angiotensin I, and then fragmented again by the angiotensin-converting enzyme (ACE) into angiotensin II (Guang et al., 2012). Angiotensin is essential for volume and blood pressure management (Almeida et al., 2020). Endothelial cells can produce angiotensin away from regulation of vascular homeostasis (RAS). Angiotensin I promoted the breakdown of bones in the coexistence of osteoclasts and osteoblast cells, according to the researchers. This study suggests that RAS could possess a function in bone breakdown regulation (Hatton et al., 1997).

Saravi et al. (2020) reported that inhibiting RAS in a laboratory animal might reduce periodontal bone degeneration and inflammatory severity. It has been found that diverse tissues and organs of rats may manufacture angiotensin separately from circulatory RAS. Surprisingly RAS is expressed regionally in rat gingival tissue, allowing for the production of angiotensin II *in vitro* (Santos et al., 2009). It could impact bone deterioration in periodontitis, although greater renin synthesis could raise periodontal vulnerability. Moreover, (Santos et al., 2009), highlighted how bacteria stimulate the production of the gingival RAS, leading to a proinflammatory microenvironment with higher angiotensin II levels, that may lead to the decline in bone density seen in periodontitis. Addressing RAS’s inflammatory function can give a new viewpoint for therapeutic studies and periodontitis therapy. RAS has been utilized in the treatment of inflammatory illnesses (Al-Azzawi et al., 2022).

#### 7.5.6 Androgens

Males’ main sexual hormone and anabolic element is testosterone. In human beings, testosterone is important in the male reproductive systems like the testes, in addition to in the stimulation of further sexual features including enhanced musculature and density of bones (Kuhn, 2002). PLGA-coated pericardial implants or membranes combined with topical progressive application of more testosterone and alendronate could be a feasible strategy for inducing local bone formation, resulting in improved implant osseous-integration and bone defect and fracture healing (van de Ven et al., 2021a). In mice, testosterone administered with a framework enhances bone formation in the same way that Bone Morphologic Protein-2 does (Cheng et al., 2013).

#### 7.5.7 Parathyroid hormone (PTH)

PTH is a significant driver of osseous remodeling in addition to being a controller of calcium-phosphate balance (Arnold et al., 2021). It stimulates osteoblasts to produce a range of growth factors while reducing osteocytes’ synthesis of sclerostin and DKK, two anti-osteoclastic and Wnt signaling antagonists. Moreover, it could have a secondary impact by encouraging osteoclasts to achieve degeneration of bones (Kikyo, 2024). PTH increases RANKL selectivity for osteoclast surface receptors while also increasing osteoblast RANKL synthesis, resulting in osteoclast activation (Sun et al., 2020).

PTH consumption has anabolic, and catabolic effects on bones. Massive quantities encourage the decomposition of bones, whereas low and irregular quantities encourage bone formation and mineral accumulation. PTH has been proven to significantly expedite fracture healing (Eastman et al., 2021). As a consequence, localized PTH supply to osseous defects could represent a viable alternative to auto transplantation (Wojda and Donahue, 2018). According to contemporary investigation, PTH improves the bony strength of the jaw and improves soft tissue repair and bone filling following exodontia (Kuroshima et al., 2013). PTH was found to possess a beneficial impact in periodontal disease animal models, by suppressing inflammation (Stutz et al., 2020). PTH dramatically decreases gingival inflammation while also suppressing bone loss. PTH administration increases bone formation via a boost in osteoblast amount, in addition to mineralized matrix accumulation via impacts on precursor division, cellular death, restriction, and lining cell stimulation, according to human and animal research (Jilka, 2007; Wein and Kronenberg, 2018).

Furthermore, investigations revealed that PTH directly promotes osteoblastic viability signaling and that the delayed death of osteoblasts is a substantial contribution to the enhanced osteoblast quantity, at least in mice (Wein and Kronenberg, 2018). Ji-Hye Kim observed that occasionally administering PTH to DM-rats with periodontitis decreased alveolar bone loss while increasing bone growth. This data implies that PTH treatment prevented bone loss caused by diabetes by stimulating bone growth. The use of the proteins SDF-1alpha and PTH increased bone growth. SDF-1alpha also promotes PDL regeneration. Multiple investigations have been conducted to investigate the effect of PTH on dental implant longevity and bone assimilation. Bellido et al.104 artificially caused osteoporosis in rabbits and assessed overall bone loss and decreased mineral levels in their jaws (Du et al., 2016). PTH injection, on the other hand, virtually totally corrected these unfavorable outcomes, restoring the jawbone to almost normal levels. Research on mongrels discovered greater amounts of bone remodeling surrounding dental implants put in the jaw in the PTH-treated group (Kim et al., 2020). A work exploring PTH-coated titanium dental implants in rats presents a possibility that may be close to usage in dentistry (Lai et al., 2017). The results showed enhanced bone growth surrounding the PTH-coated implants. As a result, our findings imply that PTH may be a potential treatment for enhancing the integration of dental implants in humans. Nevertheless, the rate of PTH delivery differs throughout research, thus determining the best interval and dose to enhance bone formation should be a top focus (Kuroshima et al., 2013). Huang et al. created an optimized delivery approach by combining a parathyroid hormone analog (PTHrP-2) with a mesoporous bioactive glass framework. In BMSCs, PTH-loaded scaffolding promoted bone formation and development. Moreover, the PTHrP-2/scaffold had decreased osteoclasts than the unaltered peptide-loaded matrix (Huang et al., 2020a). Ning et al. developed an injectable Gelatin hydrogel for sustained abaloparatide delivery. It boosted bone development and the content of minerals (Dang et al., 2017).

#### 7.5.8 Cortisol

Cortisol, a steroid hormone generated by the adrenal glands, enters the circulation, crosses the cell membrane, and translocates to attach to cell nucleus receptor proteins, causing variations in gene transcription (Chaudhuri, 2019). Cortisol is released in response to stress. Insecurity, worry, hemorrhage, discomfort, reduced blood glucose, disease, and malnutrition are common stress triggers. Because of responding to cortisol, muscle, liver, and adipose tissue deplete their nutrition store. Cortisol levels that are chronically raised cause muscle and bone degeneration, as well as compromised endocrine and immune system functionality (Stefanaki et al., 2018). Stress is a component that contributes to the beginning of sickness.

Chronic and acute stress appear to hinder tissue healing in rats. Previous research has found that prolonged stress impedes recovery, in addition to the development of the bone matrix and collagen fibers, as well as a reduction in the number of osteoblasts. Rat periodontium experimental models have revealed that anxiety enhances vulnerability and exacerbates periodontitis, PD. Several studies have found a link between stress indicators, inflammation, and periodontal disease (Warren et al., 2014). Comparably, the advancement of periodontitis is linked to stress as a cause. It has been shown that stress seems to relate to microbial colonization.

Nevertheless, salivary cortisol levels are unrelated to stress. As a result, stress and the consequent rise in cortisol could lead to the advancement of periodontitis (Siqueira et al., 2015). Cortisol inhibits parathyroid hormone’s resorptive activity, which promotes an increased number of progenitor cells and their transformation into osteoclasts. As a result, cortisol limits bone resorption *in vitro* by decreasing precursor cell capacity to produce osteoclasts.

#### 7.5.9 Insulin

Insulin is a hormonal medication for Diabetes type I (Vanea et al., 2014; Maratova et al., 2018). Insulin/IGF-1 has been shown *in vivo* to stimulate vascularity and provide nutrition for osteogenesis (Hynes et al., 2013; Paglia et al., 2013; Rabinovsky and Draghia-Akli, 2004). Insulin, by boosting the amount of bone-formation cells, may successfully promote localized skull bone development in mice (Cornish et al., 1996), and is capable of regulating osteoclastic activities (Thomas et al., 1998). Innovative insulin-loaded interactive injectable materials have been found and might be used to treat osteoarthritis, notably as a low-cost stimulator/alternative to BMP-2 approaches (Krajcer et al., 2022).

Kido et al. (2017) conducted a study on diabetic rats and discovered a link between periodontitis and diabetes. They also discovered that diabetes may trigger aberrant gingival fibroblast growth. Insulin resistance contributes to the advancement of periodontitis in diabetics. They concluded that reduced fibroblast growing and moving induced impaired gingival wound healing in diabetic rats. Fibroblast malfunction can be produced by excessive glucose-induced insulin resistance via oxidative stress. A growth factor with an architecture so close to insulin that it has been termed insulin-like growth factor-1 (IGF-1) may be produced from the liver by human growth hormone stimulation, but also by bone cells (Klein, 2014).

IGF-1 has an important role in bone formation during adolescence and throughout life. IGF-1 has also been shown in rabbits to increase bone development around dental implants when combined with other growth factors (Zhou et al., 2017; Ortolani et al., 2014). Insulin may have bone-enhancing effects with osteoblasts having insulin-receptors, possibly boosting osteoblast development, because of their similarity in architecture (Klein, 2014).

In chondrocytes, GH regulates IGF-1 production, but in osteoblasts, parathyroid hormone (PTH) regulates it. Human osteoblasts synthesize IGFs. IGF-1 stimulates developed osteoblastic function without directly altering stromal cell development into mature osteoblasts. As a result, a minimal reduction in IGF-1 expression is thought to be required for apoptosis and the transformation of osteoblasts (Javed et al., 2020).

#### 7.5.10 Estrogen

Estrogen is a naturally occurring steroid that affects the amount of bone and bone tissue homeostasis. The action of estrogen is intimately related to the control of osteoblastic multiplication and specialization. Furthermore, estrogen inhibits apoptosis in osteocytes and osteoblasts while promoting it in osteoclasts. Estrogen decreases osteoclastogenesis by lowering the generation of osteoclastic mediators. Furthermore, it stimulates the production of osteoprotegerin (OPG) by osteoblasts and osteocytes (Kearns et al., 2008).

17-estradiol (E2) attaches to estrogen receptors (ERs) in both bone cells and mesenchymal stem cells (MSCs). By increasing the activity of BMP-2, TGF-1, and IGF-1, estradiol may promote MSCs differentiation into osteoblasts and increase osteogenesis (Irmak et al., 2014). By encapsulation of E2 in an EDTA-adjusted nano composite, enhanced prolonged E2 discharge increased Alkaline phosphatase (ALP), osteopontin (OPN), osteocalcin (OCN), and calcium deposition in MC3T3-E1 preosteoblasts (Safari et al., 2021).

#### 7.5.11 Selective estrogen receptor modulators (SERMs)

SERMs are non-steroidal chemicals that possess estrogen-like impacts on the skeleton, circulatory system, and lipid levels while additionally exhibiting anti-estrogenic actions on the breast and uterine system (Ott et al., 2002; Urano et al., 2017). They stimulate endochondral ossifying, osseous production, and bone remodeling, by acting estrogenically on the skeletal skeleton (Spiro et al., 2013).

##### 7.5.11.1 Raloxifene

Raloxifene (RLX) is a selective estrogen receptor modulator (SERM), indicated to fight osteoporosis. Raloxifene has been demonstrated to have an estrogenic action on bones, in addition to increasing the preservation of bone integrity and improving bone mass density (BMD) (Ma et al., 2021). According to a lately published investigation (Zhang et al., 2017), RLX doses ranging from 0.1 to 10 g were tested *in vitro* utilizing a framework filled with PLGA microspheres carrying RLX. The findings showed that RAL discharge from microparticles was delayed and controlled, leading to improved cellular survival at all stages, significantly enhanced cellular proliferation, greater amounts of mineralized tissues, and boosted ALP functionality.

#### 7.5.12 Melatonin

The function of melatonin (ML) in hard tissues has received plenty of interest (Leonida et al., 2022; Shino et al., 2016). ML might be related to the production of hard tissues like bone and teeth (Munmun and Witt-Enderby, 2021). ML promotes tissue calcification and alkaline phosphatase function (Liu et al., 2013). As was earlier stated, ML is used for its anti-aging, anti-inflammation, and anti-free-radical capacities (Köse et al., 2017; Fernández-Ortiz et al., 2020), and cytoprotection (Dos Santos et al., 2018; Fernández-Gil et al., 2017). When there is an increased level of ML, the production of inflammatory cytokines is reduced by modifying NF-κB functions, which adds to the signaling pathway. While the beneficial effects of ML on periodontal regeneration have been demonstrated in gingival fibroblasts in addition to lab animals, additional study is required (Dos Santos et al., 2018). The circulatory half-life of ML is about 23 min (Givler et al., 2023).

ML-loaded chitosan particles can regulate Mel discharge throughout time, enhancing the osteogenic development of preosteoblast cells *in vitro* (Huang et al., 2020b). In diabetic rats, local injection of 2 mg ML gel is a promising therapy strategy for successful bone and PDL regeneration (Yousuf et al., 2013). ML appears to have the ability to be a good implant covering. When melatonin powder was administered for implantation regions in dogs, it significantly accelerated bone development and mineralization compared to control groups (Cutando et al., 2008).

### 7.6 Vitamins and antioxidants

#### 7.6.1 Vitamins

##### 7.6.1.1 Vitamin D

**FIGURE 6:**
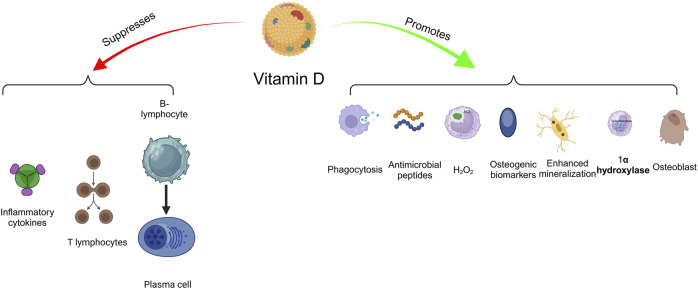
Beneficial effects of vitamin D on periodontal health.

Vitamin D is a fat-soluble molecule that exerts a crucial function in regulating osteogenesis and strength, contributing to the maintenance of calcium-phosphorus ratios in the body (Figure 6). It attaches to cells through a vitamin D receptor (VDR), modulating the expression of several genes and cell responses. It enhances the mineralization of calcified tissues, and it aids in the retention of teeth in alveolar bone by strengthening periodontal tissue.

According to research, vitamin D has an anti-inflammatory impact on periodontal tissue via several methods. Furthermore, it promotes digestive and chemotactic activity of macrophages and regulates the 1-a-hydroxylase in monocytes, which is essential to vit D production as an autocrine agent, increasing the immune reaction and being a significant element of periodontal tissue reactions to dysbiotic infections (Lu, 2023).

In terms of inflammation, vitamin D enhances the suppression of proinflammatory cytokines. It has been demonstrated that vitamin D can reduce the synthesis of cytokines such as IL-17 by T-helper (Th) cells, which has been linked to an elevated risk of periodontitis. Taskan and Gevrek discovered that those with periodontitis had a reduced amount of VDR and fewer fibroblast cells than the normal group (Taskan and Gevrek, 2020). A new *in vitro* research study demonstrated vitamin D’s anti-inflammatory and pro-mineralization benefits on periodontal tissue (Machado et al., 2020).

Periodontitis is also exacerbated by changes in the oral flora, which aggravate inflammation. It has been demonstrated to reduce the quantity of living *P. gingivalis* via activating autophagy (Hu et al., 2020). Other research indicates that elevated vitamin D concentrations reduce inflammatory amounts of cytokines such as RANKL, IL-1, and IL-6 (Han et al., 2019; Wang et al., 2020b; Li et al., 2019). Vit D has also been shown to be an important molecule in the maintenance of tooth mineralization and bone architecture.

Furthermore, vitamin D concentration was shown to be inversely associated with periodontitis severity. Numerous investigations have shown that vitamin D has a great capacity for both osteoinduction and odonto-induction. This compound has increased the levels of osteogenic biomarkers, and bone mineralization at low dosages (Mucuk et al., 2017). Bordini et al. developed a scaffold containing 1 nM 1, 25-dihydroxy vitamin D3. They observed that vitamin D3 may enhance the levels of odontoblastic markers (Bordini et al., 2020). Sattary et al. developed a polycaprolactone/gelatin framework containing HA nanoparticles recently. They observed that the incorporation of vitamin D into the scaffold blend boosts osteogenic growth and stiffening capability in hADSCs. On day 14, the combined effect of vitamin D and HA nanoparticles increased the osteogenic biomarkers in the PCL/Gel/nHA/Vit D3 scaffold group (Sattary et al., 2019).

##### 7.6.1.2 Vitamin C

Because individuals cannot produce vitamin C, which is referred to as L-ascorbic acid, it must be obtained from a healthy diet (Padayatty and Levine, 2016). Plant-based foods, which are reliable providers of this nutrient, provide around 90% of the everyday need. It has to do with the production of collagen, which is essential for giving connective tissue firmness. As for the periodontal tissues, signs involve gum discomfort, tenderness, and bleeding brought on by the vessels’ brittleness, which might eventually result in tooth loss. Vit C promotes the development of collagen (Gref et al., 2020). Because vitamin C is required for the collagen stabilization approach, its shortage may result in structural disorientation, which relates to the deterioration of periodontal ligaments and, as a result, tooth loss. Furthermore, L-ascorbic acid is essential for endothelial cell activity and support. According to investigations, it promotes the division of endothelial cells, apparently because of its potential to boost type IV collagen production (Pizzicannella et al., 2021; Bai et al., 2021). The effect of vit C in collagen tertiary architecture stabilization is also critical. Moreover, it increases the movement of fibroblasts in the epidermis and the division of keratinocytes, which might boost wound healing caused by gingivitis by lowering inflammation within the gums (Rembe et al., 2018; Pullar et al., 2017).

Numerous functions of vitamin C also regulate immunity. This chemical can shield vital biomolecules from harm caused by contaminants, harmful substances, and free radicals by providing electrons since it is a powerful antioxidant (Marconi et al., 2021). The activity of neutrophils is also significantly impacted by vitamin C, which promotes their migration toward the infection and heightens internalization and bacterial death. Additionally, it encourages neutrophils to die and be absorbed by macrophages (Carr and Maggini, 2017).

Vit C is regarded as a crucial supplemental antioxidant for periodontal health because it eliminates excessive ROS. By causing the development of periodontal ligament progenitor cells, vitamin C is also essential for avoiding and minimizing the course of periodontitis. To lessen the detrimental impacts of diabetes on periodontium, vitamin C could be used topically to treat ligature wire-induced periodontitis in diabetic rats (Toraman et al., 2020). Locally administered vit C resulted in a significant decrease in inflammatory cells and epithelial thickness, as well as an increase in the quantity of freshly generated sub basal capillaries, and was an efficient supplementary approach in treating different forms of chronic gingival inflammation (Yussif et al., 2016).

#### 7.6.2 Antioxidants

##### 7.6.2.1 Omega-3

Docosahexaenoic acid (DHA) and eicosapentaenoic acid (EPA) are two examples of omega-3 PUFA that have been demonstrated to have a variety of benefits, involving anti-inflammatory, immunoregulatory, and antioxidant-enhancing qualities. Omega-3 PUFA has been found to be curative and preventive in the management of a variety of inflammation-related disorders (Stańdo et al., 2020).

#### 7.6.3 Carnitine

L-Carnitine (L-C), a cofactor in β-oxidation of fatty acids, has been shown to promote the actions of human osteoblasts. It greatly raises osteoblast levels of collagen type I, bone sialoproteins, and osteopontin in addition to their function and multiplication (Marcovina et al., 2013; Terruzzi et al., 2019). L-C may assist in preserving the integrity of bones by preventing the effects of oxidative stress (Terruzzi et al., 2019).

#### 7.6.4 Herbal products

Natural chemicals are currently generating increased attention. The general public’s curiosity about herbal products has grown, particularly among people with chronic conditions (Alkhursani et al., 2023a; Alkhursani et al., 2023b). Products containing natural components offer extra anti-inflammatory and antioxidant characteristics that may enhance gingival health (Xue et al., 2018). Nevertheless, the variability of preparations may reduce the effectiveness of active and herbal medicines. Individual plants have mild antiseptic properties, thus mixing several herbs and chemicals may enhance their antibacterial actions. As a result, they could be utilized for the avoidance and management of early-stage periodontitis (Sadek et al., 2024).

##### 7.6.4.1 Allium sativum (garlic)

Garlic is a natural plant that may be utilized as a cost-effective and safe alternative therapy. Garlic includes allicin, which could possess antimicrobial and anti-inflammatory effects. Furthermore, garlic extract has high biocompatibility and can promote cell development. It may enhance the vitality and replication of human gingival fibroblasts (Bramanti et al., 2018). Garlic has been shown to exhibit anti-proteolytic effects against *P. gingivalis* protease, as indicated by aged garlic extract (AGE), which shows high bacteriostatic properties against *P. gingivalis* and gelatin liquefaction following 250 μL/mL dosage delivery (Shetty et al., 2013).

##### 7.6.4.2 *Aloe barbadensis* miller (aloe vera)

Aloe vera’s therapeutic benefits extend over thousands of years (Darzi et al., 2021). Aloe vera has 75 elements, including minerals, enzymes, sugars, anthraquinone, and salicylic acid (Saleem, 2021). Aloe vera possesses antibacterial properties against *Streptococcus pyogenes* and *Enterococcus faecalis* (Ghasemi et al., 2020).

It is ideal for treating gingivitis and periodontitis because it contains an anti-inflammatory ingredient (C-glucosyl chromone), inhibits the COX pathway, lowers PGE2, and degrades the bradykinin inflammatory substance involved for pain creation (Taalab et al., 2023). It helps to minimize edema, bleeding, and gingival tissue irritation. It is useful in deep pockets where normal cleaning is difficult, and its antifungal characteristics can help cure denture stomatitis, aphthous ulcerations, and angular cheilitis. Administering it following extractions is a potent healer (Bai et al., 2023).

##### 7.6.4.3 Amphipterygium adstringens

*Amphipterygium adstringens* is a Mexican indigenous plant from the Julianaceae family known as “Cuachalalate” (Beltrán-Rodríguez et al., 2021). According to recent research, the key chemical accountable for the plant’s powers is (Sotelo-Barrera et al., 2022), that possesses antioxidant, anti-inflammatory, cancer-fighting, antiulcer, and antibacterial properties (Torres-Ortiz et al., 2023).

##### 7.6.4.4 *Camellia sinensis* (green tea)

Camellia sinensis is a member of the Theaceae family, and its tiny perennial plants are frequently used to create green and black tea (Hazra et al., 2021). Green tea’s benefits are linked to its polyphenol constituents (catechins). Epicatechin-3-gallate and epigallocatechin-3 gallate are the two most important catechins. Green tea has more polyphenols (30%–40% vs. 3%–10%) than black tea, which increases antioxidant capacity and has powerful anti-inflammatory, antibacterial, antiviral, antimutagenic, and anti-aging properties (Tang et al., 2019). Green tea has a favorable effect on inflammation and periodontal disease. Thus, evidence suggests that green tea is capable of treating and avoiding periodontal disease (Liao et al., 2020).

##### 7.6.4.5 *Cinnamomum zeylanicum* (ceylon cinnamon)

Cinnamon has been utilized as an herb for cooking and in traditional medicine. Cinnamon has been examined during pregnancy, diabetic control, and gynecological diseases (Heshmati et al., 2021). It possesses anti-inflammatory, antioxidative, antimicrobial, and properties (Almatroodi et al., 2020). Cinnamon is associated with a group of over 250 evergreen trees from the Lauraceae family (Suriyagoda et al., 2021). Numerous kinds have been investigated, particularly those related to oral medicine. Cinnamomum verum and Cinnamomum zeylanicum are two of the most widely researched cinnamon varieties. Cassia cinnamon, frequently identified as Chinese cinnamon or *Cinnamomum aromaticum*, is a well-researched spice. *C. burmannii* and *C. loureiroi* are two other important cinnamon species (Bandaranayake et al., 2023; Wang et al., 2020c).

Cinnamomum bark essential oil (CBEO) is rich in aromatic chemicals, including cinnamaldehyde and eugenol. CBEO and cinnamaldehyde have antimicrobial, anti-inflammatory, and anticancer activities (Wang et al., 2020a). According to Wang et al., the cinnamaldehyde in *C. zeylanicum* bark essential oil is effective against *P. gingivalis* (Wang et al., 2018c). Based on research, cinnamaldehyde is accountable for CBEO’s antimicrobial activity (Wang et al., 2018c). The relative function of cinnamaldehyde was discovered by studying the cell microstructure, membrane integrity, and membrane characteristics (Chen et al., 2024). CBEO and cinnamaldehyde can permanently damage bacterial membranes, jeopardizing membrane functionality. When the cell membrane depolarizes, metabolism fails and bacteria die (Ivanišová, 2023). Propidium iodide uptake experiments revealed that the CBEO and cinnamaldehyde treatments damaged the bacterial membranes. The confocal microscopy investigation of *P. gingivalis* revealed PI incorporation, indicating a cell membrane rupture (Wang et al., 2018c). Microbes can be destroyed via this main method, which is called membrane damage (Meng et al., 2016). *P. gingivalis* may be vulnerable to membrane permeability produced by CBEO and cinnamaldehyde.

## 8 Future perspectives on periodontal regeneration

Periodontitis is the sixth most common chronic disease worldwide, imposing enormous costs on society and economies. Cognitive adaptation, risk factor control, and cause-related therapy have been considered the “gold standard” approach for managing periodontitis (Ren et al., 2023). Considering that host proinflammatory and immune reactions play important roles in the etiology of periodontitis and influence treatment responses, numerous adjuvant techniques have been developed to modulate host reactions and improve periodontal therapy and maintenance outcomes. We’ve already highlighted LDDS’s benefits over systemic delivery (Sholapurkar et al., 2020). Additionally, they are appropriate for diabetic patients, who frequently have other linked systemic illnesses and necessitate several medications, increasing the risk of adverse responses if systemic administration is the sole choice.

As our understanding of periodontal disease and medicine administration has advanced, tailored delivery systems have been created to assist minimize the unfavorable systemic adverse reactions of drugs. Nanomedicines are on the rise, and their incorporation into effective periodontal therapies is possible, thanks to customized local drug delivery methods (Caporossi et al., 2020). Antibiotics and anti-inflammatory drugs administered to a specific location via nanoparticle-based local drug delivery systems are more likely to be successful since they come into direct contact with biofilms or host cells. Nanotechnology has produced outstanding outcomes in this field, and its increasing use as an adjuvant has fundamentally transformed the prognoses and outcomes of standard periodontal therapy procedures (Balta et al., 2021).

As a consequence, nanocarrier technology may soon dominate the pharmaceutical business as a whole. The present situation of this expanding field indicates that its potential is essentially limitless (Chen et al., 2022). As a result of a growing comprehension of the principles of microbial activity and development of periodontitis, there has been a noteworthy shift in periodontal pocket topical delivery strategies in periodontitis treatment.

As a result, both academics and clinicians are increasingly passionate about exploring novel therapeutic techniques that may promote periodontal regeneration (Makvandi et al., 2021). To get better treatment effects, drug delivery systems must be employed correctly to release beneficial pharmaceutical agents Prospective studies should concentrate on how to tailor local medication delivery systems in order to maximize prospective clinical procedures in periodontal care.

## 9 Conclusions and prospective outlook

The topical application of repurposed medicines offers a lot of promise for modifying oral bone and periodontal healing processes. Because of their beneficial consequences, these relocated drug delivery scaffoldings are predicted to have excellent therapeutic benefits in bone and periodontal problems. Nonetheless, clinical studies for this distribution technique are now being conducted. Nevertheless, given the capacity to enhance oral bone and periodontal tissue regeneration, these delivery strategies may soon be enhanced for professional use. As it was earlier noted, there have been significant advancements in enhancing the therapeutic outcome of oral bone and periodontal treatment. Scientific advances in tissue engineering technologies, particularly in periodontium and oral bone, have provided researchers with various viable options. These advancements hold the potential for developing clinically beneficial methods that not only regenerate oral bone but also restore the periodontium, preserving its architecture and functionality.

## References

[B1] Abdel Nasser Atia G. Shalaby H. K. Zehravi M. Ghobashy M. M. Ahmad Z. Khan F. S. (2022). Locally applied repositioned hormones for oral bone and periodontal tissue engineering: a narrative review. Polymers 14, 2964. [Online]. 10.3390/polym14142964 35890740 PMC9319147

[B2] Abd Rahman F. Mohd Ali J. Abdullah M. Abu Kasim N. H. Musa S. (2016). Aspirin enhances osteogenic potential of periodontal ligament stem cells (PDLSCs) and modulates the expression profile of growth factor–associated genes in PDLSCs. J. Periodontology 87, 837–847. 10.1902/jop.2016.150610 26846966

[B3] Abedi N. Rajabi N. Kharaziha M. Nejatidanesh F. Tayebi L. J. J. O. O. B. Research C. (2022). Layered scaffolds in periodontal regeneration. J. Oral Biol. Craniofac. Res. 12, 782–797. 10.1016/j.jobcr.2022.09.001 36159068 PMC9489757

[B4] Adya R. Tan B. K. Randeva H. S. (2015). Differential effects of leptin and adiponectin in endothelial angiogenesis. J. diabetes Res. 2015, 1–12. 10.1155/2015/648239 PMC4310451 25650072

[B5] Akay A. S. ArıSAN V. Cevher E. Sessevmez M. Cam B. (2020). Oxytocin-loaded sustained-release hydrogel graft provides accelerated bone formation: an experimental rat study. J. Orthop. Res. 38, 1676–1687. 10.1002/jor.24607 32017187

[B6] Akram Z. Vohra F. Javed F. (2018). Locally delivered metformin as adjunct to scaling and root planing in the treatment of periodontal defects: a systematic review and meta-analysis. J. Periodontal Res. 53, 941–949. 10.1111/jre.12573 29858876

[B7] Al-Azzawi I. S. Mohammed N. S. Saad I. (2022). The impact of angiotensin converting enzyme-2 (ACE-2) on bone remodeling marker osteoprotegerin (OPG) in post-COVID-19 Iraqi patients. Cureus 14, e29926. 10.7759/cureus.29926 36348825 PMC9633432

[B8] Albu A. Fodor D. Bondor C. Crăciun A. M. (2013). Bone metabolism regulators and arterial stiffness in postmenopausal women. Maturitas 76, 146–150. 10.1016/j.maturitas.2013.07.001 23916080

[B9] Aleem A. R. Shahzadi L. Alvi F. Khan A. F. Chaudhry A. A. Ur Rehman I. (2017). Thyroxin releasing chitosan/collagen based smart hydrogels to stimulate neovascularization. Mater. and Des. 133, 416–425. 10.1016/j.matdes.2017.07.053

[B10] Alghonemy W. Y. Hegazy A. A. Elmigdadi F. Nasser G. A. Helal M. B. J. T. R. I. A. (2024). Potential teratogenic effect of prenatal dexamethasone administration on palate development: experimental study in rats. Transl. Res. Anat. 37, 100338. 10.1016/j.tria.2024.100338

[B11] Alkhursani S. A. Barai H. R. Alshangiti D. M. Al-Gahtany S. A. Ghobashy M. M. Atia G. A. N. (2023a). pH-sensitive hydrogel membrane-based sodium alginate/poly (vinyl alcohol) cross-linked by freeze–thawing cycles for dye water purification. ACS ES&T Water 4 **,** 509–519. 10.1021/acsestwater.3c00567

[B12] Alkhursani S. A. Ghobashy M. M. Alshangiti D. M. Al-Gahtany S. A. Meganid A. S. Nady N. (2023b). Plastic waste management and safety disinfection processes for reduced the COVID-19 Hazards. Int. J. Sustain. Eng. 16, 1–14. 10.1080/19397038.2023.2188396

[B13] Almatroodi S. A. Alsahli M. A. Almatroudi A. Anwar S. Verma A. K. Dev K. (2020). Cinnamon and its active compounds: a potential candidate in disease and tumour management through modulating various genes activity. Gene Rep. 21, 100966. 10.1016/j.genrep.2020.100966

[B14] Almeida L. F. Tofteng S. S. Madsen K. Jensen B. L. (2020). Role of the renin–angiotensin system in kidney development and programming of adult blood pressure. Clin. Sci. 134, 641–656. 10.1042/cs20190765 32219345

[B15] Arafa E.-S. A. Elgendy N. O. Elhemely M. A. Abdelaleem E. A. Mohamed W. R. J. B. (2023). Diosmin mitigates dexamethasone-induced osteoporosis *in vivo*: role of Runx2, RANKL/OPG, and oxidative stress. RANKL/OPG, oxidative stress 161, 114461. 10.1016/j.biopha.2023.114461 36889109

[B16] Arnold A. Dennison E. Kovacs C. S. Mannstadt M. Rizzoli R. Brandi M. L. (2021). Hormonal regulation of biomineralization. Nat. Rev. Endocrinol. 17, 261–275. 10.1038/s41574-021-00477-2 33727709

[B17] Atia G. A. Rashed F. Taher E. S. Cho S.-G. Dayem A. A. Soliman M. M. (2024a). Challenges of therapeutic applications and regenerative capacities of urine based stem cells in oral, and maxillofacial reconstruction. PHARMACOTHERAPY 177, 117005. 10.1016/j.biopha.2024.117005 38945084

[B18] Atia G. A. N. Mohamed S. Z. Halim H. A. Ghobashy M. M. Foda T. Shalaby H. K. (2024b). Advances in Bioceramic silicates for therapeutic, and regenerative Dentofacial reconstruction. Ceram. Int. 50, 22184–22208. 10.1016/j.ceramint.2024.04.035

[B19] Badria F. A. (2020). Drug repurposing: hypothesis, molecular aspects and therapeutic applications. IntechOpen.

[B20] Bai R. Chang Y. Saleem A. Wu F. Tian L. Zhang S. (2021). Ascorbic acid can promote the generation and expansion of neuroepithelial-like stem cells derived from hiPS/ES cells under chemically defined conditions through promoting collagen synthesis. Stem Cell Res. Ther. 12, 48–17. 10.1186/s13287-020-02115-6 33422132 PMC7796386

[B21] Bai Y. Niu Y. Qin S. Ma G. J. P. (2023). A new biomaterial derived from Aloe vera—acemannan from basic studies to clinical application. Pharmaceutics 15, 1913. 10.3390/pharmaceutics15071913 37514099 PMC10385217

[B22] Balli U. Keles G. C. Cetinkaya B. O. Mercan U. Ayas B. Erdogan D. (2014). Assessment of vascular endothelial growth factor and matrix metalloproteinase-9 in the periodontium of rats treated with atorvastatin. J. Periodontol. 85, 178–187. 10.1902/jop.2013.130018 23646851

[B23] Balta M. Papathanasiou E. Blix I. Van Dyke T. J. J. O. D. R. (2021). Host modulation and treatment of periodontal disease. J. Dent. Res. 100, 798–809. 10.1177/0022034521995157 33655803 PMC8261853

[B24] Bandaranayake P. C. Naranpanawa N. Chandrasekara C. B. Samarakoon H. Lokuge S. Jayasundara S. (2023). Chloroplast genome, nuclear ITS regions, mitogenome regions, and Skmer analysis resolved the genetic relationship among Cinnamomum species in Sri Lanka. PLoS ONE 18, e0291763. 10.1371/journal.pone.0291763 37729154 PMC10511092

[B25] Baru O. Pop L. Raduly L. Bica C. Mehterov N. Pirlog R. (2024). The evaluation of a 5-miRNA panel in patients with periodontitis disease. JDR Clin. Trans. Res. 10.1177/23800844241252395 PMC11653349 38819194

[B26] Batool F. Agossa K. Lizambard M. Petit C. Bugueno I. M. Delcourt-Debruyne E. (2019). *In-situ* forming implants loaded with chlorhexidine and ibuprofen for periodontal treatment: proof of concept study *in vivo* . Int. J. Pharm. 569, 118564. 10.1016/j.ijpharm.2019.118564 31352049

[B27] Belibasakis G. Manoil D. J. J. O. D. R. (2021). Microbial community-driven etiopathogenesis of peri-implantitis. J. Dent. Res. 100, 21–28. 10.1177/0022034520949851 32783779 PMC7754824

[B28] Beltrán-Rodríguez L. Valdez-Hernández J. I. Saynes-Vásquez A. Blancas J. Sierra-Huelsz J. A. Cristians S. (2021). Sustaining medicinal barks: survival and bark regeneration of Amphipterygium adstringens (Anacardiaceae), a tropical tree under experimental debarking. Sustainability 13, 2860. 10.3390/su13052860

[B29] Bhattacharyya S. Bhattacharyya K. Maitra A. (2012). Possible mechanisms of interaction between statins and vitamin D. Qjm 105, 487–491. 10.1093/qjmed/hcs001 22323613

[B30] Bordini E. A. F. Cassiano F. B. Silva I. S. P. Usberti F. R. Anovazzi G. Pacheco L. E. (2020). Synergistic potential of 1α, 25-dihydroxyvitamin D3 and calcium–aluminate–chitosan scaffolds with dental pulp cells. Clin. Oral Investig. 24, 663–674. 10.1007/s00784-019-02906-z 31119382

[B31] Botelho J. Machado V. Mascarenhas P. Rua J. Alves R. Cavacas M. A. (2018). Stress, salivary cortisol and periodontitis: a systematic review and meta-analysis of observational studies. Archives Oral Biol. 96, 58–65. 10.1016/j.archoralbio.2018.08.016 30189327

[B32] Bramanti I. Sudarso I. Wahyuningsih M. Wibawa T. Karina V. Kusumawardani B. J. B. J. O. M. S. (2018). Ethanolic garlic extract (Allium sativumL) increased viability and proliferation of human gingival fibroblast *in vitro* . Bangladesh J. Med. Sci. 17, 556–561. 10.3329/bjms.v17i4.38315

[B33] Breuil V. Amri E.-Z. Panaia-Ferrari P. Testa J. Elabd C. Albert-Sabonnadière C. (2011). Oxytocin and bone remodelling: relationships with neuropituitary hormones, bone status and body composition. Jt. Bone Spine 78, 611–615. 10.1016/j.jbspin.2011.02.002 21441053

[B34] Breuil V. Trojani M.-C. Ez-Zoubir A. (2021). Oxytocin and bone: review and perspectives. Int. J. Mol. Sci. 22, 8551. 10.3390/ijms22168551 34445256 PMC8395200

[B35] Calori I. R. Braga G. De Jesus P. D. C. C. Bi H. Tedesco A. C. (2020). Polymer scaffolds as drug delivery systems. Eur. Polym. J. 129, 109621. 10.1016/j.eurpolymj.2020.109621

[B36] Caporossi L. S. Dos Santos C. S. Calcia T. B. B. Cenci M. S. Muniz F. W. M. G. Da Silveira Lima G. J. C. O. I. (2020). Pharmacological management of pain after periodontal surgery: a systematic review with meta-analysis. Clin. Oral Investig. 24, 2559–2578. 10.1007/s00784-020-03401-6 32572640

[B37] Carcuac O. Berglundh T. J. J. O. D. R. (2014). Composition of human peri-implantitis and periodontitis lesions. J. Dent. Res. 93, 1083–1088. 10.1177/0022034514551754 25261052 PMC4293768

[B38] Carr A. C. Maggini S. (2017). Vitamin C and immune function. Nutrients 9, 1211. 10.3390/nu9111211 29099763 PMC5707683

[B39] Chamani S. Liberale L. Mobasheri L. Montecucco F. Al-Rasadi K. Jamialahmadi T. (2021). The role of statins in the differentiation and function of bone cells. Eur. J. Clin. Investigation 51, e13534. 10.1111/eci.13534 33656763

[B40] Chaudhuri A. (2019). Pathophysiology of stress: a review. Int. J. Res. Rev. 6, 199–213.

[B41] Chauhan N. Gupta P. Arora L. Pal D. Singh Y. (2021). Dexamethasone-loaded, injectable pullulan-poly(ethylene glycol) hydrogels for bone tissue regeneration in chronic inflammatory conditions. Mater. Sci. Eng. C 130, 112463. 10.1016/j.msec.2021.112463 34702538

[B42] Chen S. H. Chou F. F. Ko J. Y. (2010). The use of simvastatin with aromasin in an ovariectomized rat model: effects on the skeletal system. Chang. Gung Med. J. 33, 509–514. 20979701

[B43] Cheng B.-H. Chu T.-M. G. Chang C. Kang H.-Y. Huang K.-E. (2013). Testosterone delivered with a scaffold is as effective as bone morphologic protein-2 in promoting the repair of critical-size segmental defect of femoral bone in mice. PLoS One 8, e70234. 10.1371/journal.pone.0070234 23940550 PMC3733987

[B44] Chen H. Zhang Y. Yu T. Song G. Xu T. Xin T. (2022). Nano-Based drug delivery systems for periodontal tissue regeneration. Pharmaceutics 14, 2250. 10.3390/pharmaceutics14102250 36297683 PMC9612159

[B45] Chen X. Liu P. Luo X. Huang A. Wang G. J. E. F. R. TECHNOLOGY (2024). Study on the antibacterial activity and mechanism of Cinnamaldehyde against Methicillin-resistant *Staphylococcus aureus* . Study Antibact. activity Mech. Cinnamaldehyde against Methicillin-resistant Staphylococcus aureus 250, 1069–1081. 10.1007/s00217-023-04446-z

[B46] Chen Y. Kawazoe N. Chen G. (2018). Preparation of dexamethasone-loaded biphasic calcium phosphate nanoparticles/collagen porous composite scaffolds for bone tissue engineering. Acta Biomater. 67, 341–353. 10.1016/j.actbio.2017.12.004 29242161

[B47] China S. P. Sanyal S. Chattopadhyay N. (2018). Adiponectin signaling and its role in bone metabolism. Cytokine 112, 116–131. 10.1016/j.cyto.2018.06.012 29937410

[B48] Chopra I. Li H. F. Wang H. Webster K. A. (2012). Phosphorylation of the insulin receptor by AMP-activated protein kinase (AMPK) promotes ligand-independent activation of the insulin signalling pathway in rodent muscle. Diabetologia 55, 783–794. 10.1007/s00125-011-2407-y 22207502 PMC4648248

[B49] Colaianni G. Sun L. Di Benedetto A. Tamma R. Zhu L. L. Cao J. (2012). Bone marrow oxytocin mediates the anabolic action of estrogen on the skeleton. J. Biol. Chem. 287, 29159–29167. 10.1074/jbc.m112.365049 22761429 PMC3436530

[B50] Cornish J. Callon K. E. Reid I. R. (1996). Insulin increases histomorphometric indices of bone formation *in vivo* . Calcif. Tissue Int. 59, 492–495. 10.1007/s002239900163 8939777

[B51] Cutando A. Gómez‐Moreno G. Arana C. Muñoz F. Lopez‐Peña M. Stephenson J. (2008). Melatonin stimulates osteointegration of dental implants. J. Pineal Res. 45, 174–179. 10.1111/j.1600-079x.2008.00573.x 18298460

[B52] Dalcico R. De Menezes A. M. Deocleciano O. B. Oriá R. B. Vale M. L. Ribeiro R. A. (2013). Protective mechanisms of simvastatin in experimental periodontal disease. J. Periodontol. 84, 1145–1157. 10.1902/jop.2012.120114 23181416

[B53] Dang M. Koh A. J. Jin X. Mccauley L. K. Ma P. X. (2017). Local pulsatile PTH delivery regenerates bone defects via enhanced bone remodeling in a cell-free scaffold. Biomaterials 114, 1–9. 10.1016/j.biomaterials.2016.10.049 27835763 PMC5125900

[B54] Darzi S. Paul K. Leitan S. Werkmeister J. A. Mukherjee S. J. I. J. O. M. S. (2021). Immunobiology and application of aloe vera-based scaffolds in tissue engineering. Int. J. Mol. Sci. 22, 1708. 10.3390/ijms22041708 33567756 PMC7915752

[B55] De Araújo Júnior R. F. Souza T. O. De Moura L. M. Torres K. P. De Souza L. B. Alves Mdo S. (2013). Atorvastatin decreases bone loss, inflammation and oxidative stress in experimental periodontitis. PLoS One 8, e75322. 10.1371/journal.pone.0075322 24130702 PMC3794930

[B56] De Giorgi R. Rizzo Pesci N. Rosso G. Maina G. Cowen P. J. Harmer C. J. J. T. P. (2023). The pharmacological bases for repurposing statins in depression: a review of mechanistic studies. Transl. Psychiatry 13, 253. 10.1038/s41398-023-02533-z 37438361 PMC10338465

[B57] Díaz‐González F. Sánchez‐Madrid F. (2015). NSAIDs: learning new tricks from old drugs. Eur. J. Immunol. 45, 679–686. 10.1002/eji.201445222 25523026 PMC5065088

[B58] Diaz P. Gonzalo E. Villagra L. J. G. Miegimolle B. Suarez M. J. J. B. O. H. (2022). What is the prevalence of peri-implantitis? A systematic review and meta-analysis. BMC Oral Health 22, 449. 10.1186/s12903-022-02493-8 36261829 PMC9583568

[B59] Dos Santos R. M. Marani F. Chiba F. Y. Mattera M. S. D. L. C. Tsosura T. V. S. Tessarin G. W. L. (2018). Melatonin promotes reduction in TNF levels and improves the lipid profile and insulin sensitivity in pinealectomized rats with periodontal disease. Life Sci. 213, 32–39. 10.1016/j.lfs.2018.09.056 30321542

[B60] Du J. Mei S. Guo L. Su Y. Wang H. Liu Y. (2018a). Platelet-rich fibrin/aspirin complex promotes alveolar bone regeneration in periodontal defect in rats. J. Periodontal Res. 53, 47–56. 10.1111/jre.12485 28862325

[B61] Du J. Mei S. Guo L. Su Y. Wang H. Liu Y. (2018b). Platelet-rich fibrin/aspirin complex promotes alveolar bone regeneration in periodontal defect in rats. J. Periodontal Res. 53, 47–56. 10.1111/jre.12485 28862325

[B62] Du L. Feng R. Ge S. (2016). PTH/SDF‐1α cotherapy promotes proliferation, migration and osteogenic differentiation of human periodontal ligament stem cells. Cell Prolif. 49, 599–608. 10.1111/cpr.12286 27523567 PMC6496697

[B63] Eastman K. Gerlach M. Piec I. Greeves J. Fraser W. J. O. I. (2021). Effectiveness of parathyroid hormone (PTH) analogues on fracture healing. a meta-analysis, 1–16. 10.1007/s00198-021-05847-0 33559713

[B64] Elavarasu S. Suthanthiran T. K. Naveen D. (2012). Statins: a new era in local drug delivery. J. Pharm. Bioallied Sci. 4, S248–S251. 10.4103/0975-7406.100225 23066263 PMC3467872

[B65] El-Nablaway M. Rashed F. Taher E. S. Atia G. A. Foda T. Mohammed N. A. et al. (2024a). Bioactive injectable mucoadhesive thermosensitive natural polymeric hydrogels for oral bone and periodontal regeneration. Front. Bioeng. Biotechnol., 12, 1384326. 10.3389/fbioe.2024.1384326 38863491 PMC11166210

[B66] El-Nablaway M. Rashed F. Taher E. S. Foda T. Abdeen A. Abdo M. (2024b). Prospectives and challenges of nano-tailored biomaterials-assisted biological molecules delivery for tissue engineering purposes. Life Sci. 349, 122671. 10.1016/j.lfs.2024.122671 38697279

[B67] Ertugrul D. T. Yavuz B. Cil H. Ata N. Akin K. O. Kucukazman M. (2011). STATIN-D study: comparison of the influences of rosuvastatin and fluvastatin treatment on the levels of 25 hydroxyvitamin D. Cardiovasc Ther. 29, 146–152. 10.1111/j.1755-5922.2010.00141.x 20370794

[B68] Fang H. Judd R. L. (2018). Adiponectin regulation and function. Compr. Physiol. 8, 1031–1063. 10.1002/cphy.c170046 29978896

[B69] Farooq A. Yar M. Khan A. S. Shahzadi L. Siddiqi S. A. Mahmood N. (2015). Synthesis of piroxicam loaded novel electrospun biodegradable nanocomposite scaffolds for periodontal regeneration. Mater Sci. Eng. C Mater Biol. Appl. 56, 104–113. 10.1016/j.msec.2015.06.006 26249571

[B70] Feixiang L. Yanchen F. Xiang L. Yunke Z. Jinxin M. Jianru W. (2023). The mechanism of oxytocin and its receptors in regulating cells in bone metabolism. Front. Pharmacol. 14, 1171732. 10.3389/fphar.2023.1171732 37229246 PMC10203168

[B71] Fernández-Gil B. Moneim A. E. A. Ortiz F. Shen Y.-Q. Soto-Mercado V. Mendivil-Perez M. (2017). Melatonin protects rats from radiotherapy-induced small intestine toxicity. PloS one 12, e0174474. 10.1371/journal.pone.0174474 28403142 PMC5389624

[B72] Fernández-Ortiz M. Sayed R. K. A. Fernández-Martínez J. Cionfrini A. Aranda-Martínez P. Escames G. (2020). Melatonin/Nrf2/NLRP3 connection in mouse heart mitochondria during aging. Antioxidants 9, 1187. 10.3390/antiox9121187 33260800 PMC7760557

[B73] Fraser D. Caton J. Benoit D. S. J. F. I. D. M. (2022). Periodontal wound healing and regeneration: insights for engineering new therapeutic approaches. Front. Dent. Med. 3, 815810. 10.3389/fdmed.2022.815810

[B74] Freeman F. E. Pitacco P. Van Dommelen L. H. Nulty J. Browe D. C. Shin J.-Y. (2021). Development of a 3D bioprinted scaffold with spatio-temporally defined patterns of BMP-2 and VEGF for the regeneration of large bone defects. Bio. Protoc. 11, e4219. 10.21769/bioprotoc.4219 PMC8595425 34859133

[B75] Friedrichsen M. Mortensen B. Pehmøller C. Birk J. B. Wojtaszewski J. F. P. (2013). Exercise-induced AMPK activity in skeletal muscle: role in glucose uptake and insulin sensitivity. Mol. Cell. Endocrinol. 366, 204–214. 10.1016/j.mce.2012.06.013 22796442

[B76] Fukui T. II M. Shoji T. Matsumoto T. Mifune Y. Kawakami Y. (2012). Therapeutic effect of local administration of low-dose simvastatin-conjugated gelatin hydrogel for fracture healing. J. Bone Min. Res. 27, 1118–1131. 10.1002/jbmr.1558 22275312

[B77] Fu S. Yang X. J. J. O. M. C. B. (2023). Recent advances in natural small molecules as drug delivery systems. J. Mater. Chem. B 11, 4584–4599. 10.1039/d3tb00070b 37084077

[B78] Ge B. Liu H. Liang Q. Shang L. Wang T. Ge S. (2019). Oxytocin facilitates the proliferation, migration and osteogenic differentiation of human periodontal stem cells *in vitro* . Arch. Oral Biol. 99, 126–133. 10.1016/j.archoralbio.2019.01.007 30682715

[B79] Ghasemi N. Behnezhad M. Asgharzadeh M. Zeinalzadeh E. Kafil H. S. J. I. J. O. D. (2020). Antibacterial properties of aloe vera on intracanal medicaments against *Enterococcus faecalis* biofilm at different stages of development. Int. J. Dent. 2020, 1–6. 10.1155/2020/8855277 PMC7803133 33488716

[B80] Givler D. Givler A. Luther P. M. Wenger D. M. Ahmadzadeh S. Shekoohi S. (2023). Chronic administration of melatonin: physiological and clinical considerations. Neurol. Int. 15, 518–533. 10.3390/neurolint15010031 36976674 PMC10053496

[B81] Gref R. Deloménie C. Maksimenko A. Gouadon E. Percoco G. Lati E. (2020). Vitamin C–squalene bioconjugate promotes epidermal thickening and collagen production in human skin. Sci. Rep. 10, 16883. 10.1038/s41598-020-72704-1 33037252 PMC7547010

[B82] Guang C. Phillips R. D. Jiang B. Milani F. (2012). Three key proteases–angiotensin-I-converting enzyme (ACE), ACE2 and renin–within and beyond the renin-angiotensin system. Archives Cardiovasc. Dis. 105, 373–385. 10.1016/j.acvd.2012.02.010 PMC7102827 22800722

[B83] Ha N. N.-Y. Huynh T. K. T. Phan N. U. P. Nguyen T.-H. Vong L. B. Trinh N.-T. J. R. T. (2024). Synergistic effect of metformin and vitamin D3 on osteogenic differentiation of human adipose tissue-derived mesenchymal stem cells under high D-glucose conditions. Regen. Ther. 25, 147–156. 10.1016/j.reth.2023.12.003 38486821 PMC10937201

[B84] Han J. Cheng C. Zhu Z. Lin M. Zhang D.-X. Wang Z.-M. (2019). Vitamin D reduces the serum levels of inflammatory cytokines in rat models of periodontitis and chronic obstructive pulmonary disease. J. oral Sci. 61, 53–60. 10.2334/josnusd.17-0357 30918217

[B85] Hasturk H. Kantarci A. J. P. (2015). Activation and resolution of periodontal inflammation and its systemic impact. Periodontol. 2000 69, 255–273. 10.1111/prd.12105 26252412 PMC4530469

[B86] Hatton R. Stimpel M. Chambers T. J. (1997). Angiotensin II is generated from angiotensin I by bone cells and stimulates osteoclastic bone resorption *in vitro* . J. Endocrinol. 152, 5–10. 10.1677/joe.0.1520005 9014834

[B87] Hazra A. Mahadani P. Das S. Bhattacharya S. Kumar R. Sengupta C. (2021). Insight to the ancestral relations and varietal diversity of Indian tea [Camellia sinensis (L.) Kuntze] through plastid and nuclear phylogenetic markers. Genet. Resour. Crop Evol. 68, 773–783. 10.1007/s10722-020-01022-2

[B88] Hennessy E. Adams C. Reen F. J. O'Gara F. J. A. A. (2016). Is there potential for repurposing statins as novel antimicrobials? Antimicrob. Agents Chemother. 60, 5111–5121. 10.1128/aac.00192-16 27324773 PMC4997871

[B89] Herrmann J. M. Meyle J. (2015). Neutrophil activation and periodontal tissue injury. Periodontol. 2000 69, 111–127. 10.1111/prd.12088 26252405

[B90] Heshmati J. Sepidarkish M. Morvaridzadeh M. Farsi F. Tripathi N. Razavi M. (2021). The effect of cinnamon supplementation on glycemic control in women with polycystic ovary syndrome: a systematic review and meta-analysis. J. Food Biochem. 45, e13543. 10.1111/jfbc.13543 33111340

[B91] Hong W. Wei Z. Qiu Z. Li Z. Fu C. Ye Z. (2020). Atorvastatin promotes bone formation in aged apoE–/–mice through the Sirt1–Runx2 axis. J. Orthop. Surg. Res. 15, 303–309. 10.1186/s13018-020-01841-0 32762716 PMC7412819

[B92] Huang C.-C. Law Y.-Y. Liu S.-C. Hu S.-L. Lin J.-A. Chen C.-J. (2021). Adiponectin promotes VEGF expression in rheumatoid arthritis synovial fibroblasts and induces endothelial progenitor cell angiogenesis by inhibiting miR-106a-5p. Cells 10, 2627. 10.3390/cells10102627 34685605 PMC8534315

[B93] Huang R. Y. Hsiao P. Y. Mau L. P. Tsai Y. C. Cochran D. L. Weng P. W. (2020c). Synthesis and characterization of melatonin-loaded chitosan microparticles promote differentiation and mineralization in preosteoblastic cells. J. Oral Implantol. 46, 562–570. 10.1563/aaid-joi-d-19-00208 32838427

[B94] Huang R.-Y. Hsiao P.-Y. Mau L.-P. Tsai Y.-W. C. Cochran D. L. Weng P.-W. (2020b). Synthesis and characterization of melatonin-loaded chitosan microparticles promote differentiation and mineralization in preosteoblastic cells. J. Oral Implant. 46, 562–570. 10.1563/aaid-joi-d-19-00208 32838427

[B95] Huang J. Lin D. Wei Z. Li Q. Zheng J. Zheng Q. (2020a). Parathyroid hormone derivative with reduced osteoclastic activity promoted bone regeneration via synergistic bone remodeling and angiogenesis. Small 16, e1905876. 10.1002/smll.201905876 31962381

[B96] Hulbert A. J. (2000). Thyroid hormones and their effects: a new perspective. Biol. Rev. 75, 519–631. 10.1017/s146479310000556x 11117200

[B97] Hu X. Niu L. Ma C. Huang Y. Yang X. Shi Y. (2020). Calcitriol decreases live Porphyromonas gingivalis internalized into epithelial cells and monocytes by promoting autophagy. J. Periodontology 91, 956–966. 10.1002/jper.19-0510 31774177

[B98] Hynes B. Kumar A. H. O'Sullivan J. Klein Buneker C. Leblond A. L. Weiss S. (2013). Potent endothelial progenitor cell-conditioned media-related anti-apoptotic, cardiotrophic, and pro-angiogenic effects post-myocardial infarction are mediated by insulin-like growth factor-1. Eur. Heart J. 34, 782–789. 10.1093/eurheartj/ehr435 22173909

[B99] Irmak G. Demirtaş T. T. Çetin AltıNDAL D. ÇalıŞ M. Gümüşderelioğlu M. (2014). Sustained release of 17β-estradiol stimulates osteogenic differentiation of adipose tissue-derived mesenchymal stem cells on chitosan-hydroxyapatite scaffolds. Cells Tissues Organs 199, 37–50. 10.1159/000362362 25115579

[B100] Isik G. Hasirci N. Tezcaner A. Kiziltay A. J. J. O. B. Polymers C. (2020). Multifunctional periodontal membrane for treatment and regeneration purposes. Dent. Clin. North Am. 35, 117–138. 10.1177/0883911520911659

[B101] IşıLAY Özdoğan A. Akca G. Şenel S. (2018). Development and *in vitro* evaluation of chitosan based system for local delivery of atorvastatin for treatment of periodontitis. Eur. J. Pharm. Sci. 124, 208–216. 10.1016/j.ejps.2018.08.037 30171985

[B102] Ivanišová E. (2023). Herbs and spices: new advances. IntechOpen.

[B103] Iwayama T. Yanagita M. Mori K. Sawada K. Ozasa M. Kubota M. (2012). Adiponectin regulates functions of gingival fibroblasts and periodontal ligament cells. J. Periodontal Res. 47, 563–571. 10.1111/j.1600-0765.2012.01467.x 22339084

[B104] Jain N. Jain G. K. Javed S. Iqbal Z. Talegaonkar S. Ahmad F. J. (2008). Recent approaches for the treatment of periodontitis. Drug Discov. Today 13, 932–943. 10.1016/j.drudis.2008.07.010 18789399

[B105] James H. P. John R. Alex A. Anoop K. J. A. P. S. B. (2014). Smart polymers for the controlled delivery of drugs–a concise overview. Acta Pharm. Sin. B 4, 120–127. 10.1016/j.apsb.2014.02.005 26579373 PMC4590297

[B106] Javed F. Akram Z. Khan J. Zafar M. S. (2020). “Growth factors and guided bone regeneration,” in Dental implants. Elsevier.

[B107] Jee W. S. S. Ma Y. F. (1997). The *in vivo* anabolic actions of prostaglandins in bone. Bone 21, 297–304. 10.1016/s8756-3282(97)00147-6 9315332

[B108] Jhinger N. Kapoor D. Jain R. (2015). Comparison of Periochip (chlorhexidine gluconate 2.5 mg) and Arestin (Minocycline hydrochloride 1 mg) in the management of chronic periodontitis. Indian J. Dent. 6, 20. 10.4103/0975-962x.151697 25767356 PMC4357074

[B109] Jiang M. Qi L. Li L. Wu Y. Song D. Li Y. J. I. J. O. C. (2021). Caspase‐8: a key protein of cross‐talk signal way in “PANoptosis” in cancer. cancer 149, 1408–1420. 10.1002/ijc.33698 34028029

[B110] Jiang X. Hao X. Jing L. Wu G. Kang D. Liu X. (2019). Recent applications of click chemistry in drug discovery. Expert Opin. Drug Discov. 14, 779–789. 10.1080/17460441.2019.1614910 31094231

[B111] Jiang X. Song D. Ye B. Wang X. Song G. Yang S. (2011). Effect of intermittent administration of adiponectin on bone regeneration following mandibular osteodistraction in rabbits. J. Orthop. Res. 29, 1081–1085. 10.1002/jor.21355 21344499

[B112] Jilka R. L. (2007). Molecular and cellular mechanisms of the anabolic effect of intermittent PTH. Bone 40, 1434–1446. 10.1016/j.bone.2007.03.017 17517365 PMC1995599

[B113] Jin J. Machado E. R. Yu H. Zhang X. Lu Z. Li Y. (2014a). Simvastatin inhibits LPS-induced alveolar bone loss during metabolic syndrome. J. Dent. Res. 93, 294–299. 10.1177/0022034513516980 24352501 PMC3929976

[B114] Jin J. Zhang X. Lu Z. Li Y. Lopes-Virella M. F. Yu H. (2014b). Simvastatin inhibits lipopolysaccharide-induced osteoclastogenesis and reduces alveolar bone loss in experimental periodontal disease. J. Periodontal Res. 49, 518–526. 10.1111/jre.12132 24117880 PMC3979522

[B115] Jørgensen N. R. Henriksen Z. Sørensen O. H. Civitelli R. (2004). Dexamethasone, BMP-2, and 1,25-dihydroxyvitamin D enhance a more differentiated osteoblast phenotype: validation of an *in vitro* model for human bone marrow-derived primary osteoblasts. Steroids 69, 219–226. 10.1016/j.steroids.2003.12.005 15183687

[B116] Jourdan J. P. Bureau R. Rochais C. Dallemagne P. (2020). Drug repositioning: a brief overview. J. Pharm. Pharmacol. 72, 1145–1151. 10.1111/jphp.13273 32301512 PMC7262062

[B117] Kagami S. Owada T. Kanari H. Saito Y. Suto A. Ikeda K. (2009). Protein geranylgeranylation regulates the balance between Th17 cells and Foxp3+ regulatory T cells. Int. Immunol. 21, 679–689. 10.1093/intimm/dxp037 19380384

[B118] Kamińska M. Aliko A. Hellvard A. Bielecka E. Binder V. Marczyk A. (2019). Effects of statins on multispecies oral biofilm identify simvastatin as a drug candidate targeting Porphyromonas gingivalis. J. Periodontol. 90, 637–646. 10.1002/jper.18-0179 30506795 PMC6545270

[B119] Kearns A. E. Khosla S. Kostenuik P. J. (2008). Receptor activator of nuclear factor κB ligand and osteoprotegerin regulation of bone remodeling in health and disease. Endocr. Rev. 29, 155–192. 10.1210/er.2007-0014 18057140 PMC2528846

[B120] Kido D. Mizutani K. Takeda K. Mikami R. Matsuura T. Iwasaki K. (2017). Impact of diabetes on gingival wound healing via oxidative stress. PLoS One 12, e0189601. 10.1371/journal.pone.0189601 29267310 PMC5739411

[B121] Kikyo N. J. I. J. O. M. S. (2024). Circadian regulation of bone remodeling. Int. J. Mol. Sci. 25, 4717. 10.3390/ijms25094717 38731934 PMC11083221

[B122] Kim J. Kim H.-Y. Kim W.-H. Kim J.-W. Kim M.-J. (2020). Effect of PTH and corticotomy on implant movement under mechanical force. BMC Oral Health 20, 1–10. 10.1186/s12903-020-01310-4 PMC7653691 33172437

[B123] Klein G. L. (2014). Insulin and bone: recent developments. World J. diabetes 5, 14. 10.4239/wjd.v5.i1.14 24567798 PMC3932424

[B124] Köse O. Arabaci T. Kizildag A. Erdemci B. Özkal Eminoğlu D. Gedikli S. (2017). Melatonin prevents radiation‐induced oxidative stress and periodontal tissue breakdown in irradiated rats with experimental periodontitis. J. periodontal Res. 52, 438–446. 10.1111/jre.12409 27510437

[B125] Koushki K. Shahbaz S. K. Mashayekhi K. Sadeghi M. Zayeri Z. D. Taba M. Y. (2021). Anti-inflammatory action of statins in cardiovascular disease: the role of inflammasome and toll-like receptor pathways. Clin. Rev. Allergy and Immunol. 60, 175–199. 10.1007/s12016-020-08791-9 32378144 PMC7985098

[B126] Krajcer A. Klara J. Horak W. Lewandowska-Łańcucka J. (2022). Bioactive injectable composites based on insulin-functionalized silica particles reinforced polymeric hydrogels for potential applications in bone tissue engineering. J. Mater. Sci. and Technol. 105, 153–163. 10.1016/j.jmst.2021.08.003

[B127] Kuhn C. M. (2002). Anabolic steroids. Recent Prog. hormone Res. 57, 411–434. 10.1210/rp.57.1.411 12017555

[B128] Kumari M. Martande S. S. Pradeep A. R. Naik S. B. (2016). Efficacy of subgingivally delivered 1.2% atorvastatin in the treatment of chronic periodontitis in patients with type 2 diabetes mellitus: a randomized controlled clinical trial. J. Periodontology 87, 1278–1285. 10.1902/jop.2016.130227 27442085

[B129] Kuroshima S. Kovacic B. L. Kozloff K. M. Mccauley L. K. Yamashita J. (2013). Intra-oral PTH administration promotes tooth extraction socket healing. J. Dent. Res. 92, 553–559. 10.1177/0022034513487558 23611925 PMC3654759

[B130] Lai K. XI Y. Miao X. Jiang Z. Wang Y. Wang H. (2017). PTH coatings on titanium surfaces improved osteogenic integration by increasing expression levels of BMP-2/Runx2/Osterix. Rsc Adv. 7, 56256–56265. 10.1039/c7ra09738g

[B131] Lee B.-S. Lee C.-C. Lin H.-P. Shih W.-A. Hsieh W.-L. Lai C.-H. (2016a). A functional chitosan membrane with grafted epigallocatechin-3-gallate and lovastatin enhances periodontal tissue regeneration in dogs. Carbohydr. Polym. 151, 790–802. 10.1016/j.carbpol.2016.06.026 27474626

[B132] Lee B. S. Lee C. C. Lin H. P. Shih W. A. Hsieh W. L. Lai C. H. (2016b). A functional chitosan membrane with grafted epigallocatechin-3-gallate and lovastatin enhances periodontal tissue regeneration in dogs. Carbohydr. Polym. 151, 790–802. 10.1016/j.carbpol.2016.06.026 27474626

[B133] Lee Y.-S. Kim Y.-S. Lee S.-Y. Kim G.-H. Kim B.-J. Lee S.-H. (2010). AMP kinase acts as a negative regulator of RANKL in the differentiation of osteoclasts. Bone 47, 926–937. 10.1016/j.bone.2010.08.001 20696287

[B134] Leonida A. Favero G. Caccianiga P. Ceraulo S. Rodella L. F. Rezzani R. (2022). Concentrated growth factors (cgf) combined with melatonin in guided bone regeneration (gbr): a case report. Diagnostics 12, 1257. 10.3390/diagnostics12051257 35626412 PMC9141849

[B135] Li X.-H. Pang W.-W. Zhang Y. Liu D.-Y. Yi Q.-R. Wang N. (2023). A Mendelian randomization study for drug repurposing reveals bezafibrate and fenofibric acid as potential osteoporosis treatments. Front. Pharmacol. 14, 1211302. 10.3389/fphar.2023.1211302 37547327 PMC10397407

[B136] Liang J. Peng X. Zhou X. Zou J. Cheng L. (2020). Emerging applications of drug delivery systems in oral infectious diseases prevention and treatment. Molecules 25, 516. 10.3390/molecules25030516 31991678 PMC7038021

[B137] Liao S. Tang Y. Chu C. Lu W. Baligen B. Man Y. (2020). Application of green tea extracts epigallocatechin‐3-gallate in dental materials: recent progress and perspectives. J. Biomed. Mater. Res. A 108, 2395–2408. 10.1002/jbm.a.36991 32379385

[B138] Li H. Zhong X. Li W. Wang Q. (2019). Effects of 1, 25-dihydroxyvitamin D 3 on experimental periodontitis and AhR/NF-κB/NLRP3 inflammasome pathway in a mouse model. J. Appl. Oral Sci. 27, e20180713. 10.1590/1678-7757-2018-0713 31691738 PMC6831029

[B139] Li L. Zhou G. Wang Y. Yang G. Ding S. Zhou S. (2015). Controlled dual delivery of BMP-2 and dexamethasone by nanoparticle-embedded electrospun nanofibers for the efficient repair of critical-sized rat calvarial defect. Biomaterials 37, 218–229. 10.1016/j.biomaterials.2014.10.015 25453952

[B140] Limoee M. Moradipour P. Godarzi M. Arkan E. Behbood L. (2019). Fabrication and *in-vitro* investigation of polycaprolactone - (Polyvinyl alcohol/collagen) hybrid nanofiber as anti-inflammatory guided tissue regeneration membrane. Curr. Pharm. Biotechnol. 20, 1122–1133. 10.2174/1389201020666190722161004 31333124

[B141] Liu J. Zhou H. Fan W. Dong W. Fu S. He H. (2013). Melatonin influences proliferation and differentiation of rat dental papilla cells *in vitro* and dentine formation *in vivo* by altering mitochondrial activity. J. pineal Res. 54, 170–178. 10.1111/jpi.12002 22946647 PMC3597977

[B142] Liu Y. Wang L. Kikuiri T. Akiyama K. Chen C. Xu X. (2011). Mesenchymal stem cell-based tissue regeneration is governed by recipient T lymphocytes via IFN-γ and TNF-α. Nat. Med. 17, 1594–1601. 10.1038/nm.2542 22101767 PMC3233650

[B143] Lu E. M. C. (2023). The role of vitamin D in periodontal health and disease. J. Periodontal Res. 58, 213–224. 10.1111/jre.13083 36537578

[B144] Ma H.-Y. Chen S. Lu L.-L. Gong W. Zhang A.-H. J. H. Research M. (2021). Raloxifene in the treatment of osteoporosis in postmenopausal women with end-stage renal disease: a systematic review and meta-analysis. Horm. Metab. Res. 53, 730–737. 10.1055/a-1655-4362 34740274

[B145] Machado V. Lobo S. Proença L. Mendes J. J. Botelho J. (2020). Vitamin D and periodontitis: a systematic review and meta-analysis. Nutrients 12, 2177. 10.3390/nu12082177 32708032 PMC7468917

[B146] Madhumathi K. Rubaiya Y. Doble M. Venkateswari R. Sampath Kumar T. S. (2018). Antibacterial, anti-inflammatory, and bone-regenerative dual-drug-loaded calcium phosphate nanocarriers—*in vitro* and *in vivo* studies. Drug Deliv. Transl. Res. 8, 1066–1077. 10.1007/s13346-018-0532-6 29717475

[B147] Makvandi P. Josic U. Delfi M. Pinelli F. Jahed V. Kaya E. (2021). Drug delivery (nano) platforms for oral and dental applications: tissue regeneration, infection control, and cancer management. Adv. Sci. (Weinh). 8, 2004014. 10.1002/advs.202004014 33898183 PMC8061367

[B148] Malik M. H. Shahzadi L. Batool R. Safi S. Z. Khan A. S. Khan A. F. (2020). Thyroxine-loaded chitosan/carboxymethyl cellulose/hydroxyapatite hydrogels enhance angiogenesis in in-ovo experiments. Int. J. Biol. Macromol. 145, 1162–1170. 10.1016/j.ijbiomac.2019.10.043 31730970

[B149] Maratova K. Soucek O. Matyskova J. Hlavka Z. Petruzelkova L. Obermannova B. (2018). Muscle functions and bone strength are impaired in adolescents with type 1 diabetes. Bone 106, 22–27. 10.1016/j.bone.2017.10.005 29017892

[B150] Marconi G. D. Fonticoli L. Guarnieri S. Cavalcanti M. F. Franchi S. Gatta V. (2021). Ascorbic acid: a new player of epigenetic regulation in LPS‐*gingivalis* treated human periodontal ligament stem cells. stem cells 2021, 6679708. 10.1155/2021/6679708 PMC7840256 33542783

[B151] Marcovina S. M. Sirtori C. Peracino A. Gheorghiade M. Borum P. Remuzzi G. (2013). Translating the basic knowledge of mitochondrial functions to metabolic therapy: role of L-carnitine. Transl. Res. 161, 73–84. 10.1016/j.trsl.2012.10.006 23138103 PMC3590819

[B152] Martins A. Duarte A. R. Faria S. Marques A. P. Reis R. L. Neves N. M. (2010). Osteogenic induction of hBMSCs by electrospun scaffolds with dexamethasone release functionality. Biomaterials 31, 5875–5885. 10.1016/j.biomaterials.2010.04.010 20452016

[B153] Mausner-Fainberg K. Luboshits G. Mor A. Maysel-Auslender S. Rubinstein A. Keren G. (2008). The effect of HMG-CoA reductase inhibitors on naturally occurring CD4+CD25+ T cells. Atherosclerosis 197, 829–839. 10.1016/j.atherosclerosis.2007.07.031 17826781

[B154] Mciver L. A. Siddique M. S. (2023). “Atorvastatin. *StatPearls.* Treasure island (FL) ineligible companies,” in Disclosure: momin Siddique declares no relevant financial relationships with ineligible companies. StatPearls Publishing.

[B155] Mealy J. E. Rodell C. B. Burdick J. A. J. J. O. M. C. B. (2015). Sustained small molecule delivery from injectable hyaluronic acid hydrogels through host–guest mediated retention. J. Mater. Chem. B 3, 8010–8019. 10.1039/c5tb00981b 26693019 PMC4675358

[B156] Meng X. Li D. Zhou D. Wang D. Liu Q. Fan S. J. J. O. E. (2016). Chemical composition, antibacterial activity and related mechanism of the essential oil from the leaves of Juniperus rigida Sieb. et Zucc against *Klebsiella pneumoniae* . Zucc against Klebsiella pneumoniae. 194, 698–705. 10.1016/j.jep.2016.10.050 27769947

[B157] Messora M. R. Apolinário Vieira G. H. Vanderlei J. Mariguela V. C. Fernandes P. G. Palioto D. B. (2017). Rosuvastatin promotes benefits on induced periodontitis in hypertensive rats. J. Periodontal Res. 52, 734–744. 10.1111/jre.12442 28256038

[B158] Meyle J. Chapple I. (2015). Molecular aspects of the pathogenesis of periodontitis. Periodontol. 2000 69, 7–17. 10.1111/prd.12104 26252398

[B159] Mirzaeei S. Ezzati A. Mehrandish S. Asare-Addo K. Nokhodchi A. J. J. O. D. D. S. (2022). An overview of guided tissue regeneration (GTR) systems designed and developed as drug carriers for management of periodontitis. J. Drug Deliv. Sci. Technol. 71, 103341. 10.1016/j.jddst.2022.103341

[B160] Mohamed W. (2024). Nanocarriers in neurodegenerative disorders: therapeutic hopes and hypes. Taylor and Francis group.

[B161] Monje A. Pons R. Amerio E. Wang H. L. Nart J. J. J. O. P. (2022). Resolution of peri‐implantitis by means of implantoplasty as adjunct to surgical therapy: a retrospective study. J. Periodontol. 93, 110–122. 10.1002/jper.21-0103 33904175

[B162] Moriyama Y. Ayukawa Y. Ogino Y. Atsuta I. Koyano K. (2008). Topical application of statin affects bone healing around implants. Clin. Oral Implants Res. 19, 600–605. 10.1111/j.1600-0501.2007.01508.x 18422989

[B163] Moshiri A. Shahrezaee M. Shekarchi B. Oryan A. Azma K. (2015). Three-dimensional porous gelapin–simvastatin scaffolds promoted bone defect healing in rabbits. Calcif. tissue Int. 96, 552–564. 10.1007/s00223-015-9981-9 25804980

[B164] Mu C. Hu Y. Huang L. Shen X. Li M. Li L. (2018). Sustained raloxifene release from hyaluronan-alendronate-functionalized titanium nanotube arrays capable of enhancing osseointegration in osteoporotic rabbits. Mater Sci. Eng. C Mater Biol. Appl. 82, 345–353. 10.1016/j.msec.2017.08.056 29025668

[B165] Mucuk G. Sepet E. Erguven M. Ekmekcı O. BıLıR A. (2017). 1, 25-Dihydroxyvitamin D3 stimulates odontoblastic differentiation of human dental pulp-stem cells *in vitro* . Connect. Tissue Res. 58, 531–541. 10.1080/03008207.2016.1264395 27905856

[B166] Mundy G. Garrett R. Harris S. Chan J. Chen D. Rossini G. (1999). Stimulation of bone formation *in vitro* and in rodents by statins. Science 286, 1946–1949. 10.1126/science.286.5446.1946 10583956

[B167] Munmun F. Witt‐Enderby P. A. J. J. O. P. R. (2021). Melatonin effects on bone: implications for use as a therapy for managing bone loss. J. Pineal Res. 71, e12749. 10.1111/jpi.12749 34085304

[B168] Murk A. J. Rijntjes E. Blaauboer B. J. Clewell R. Crofton K. M. Dingemans M. M. L. (2013). Mechanism-based testing strategy using *in vitro* approaches for identification of thyroid hormone disrupting chemicals. Toxicol. vitro 27, 1320–1346. 10.1016/j.tiv.2013.02.012 23453986

[B169] Nasiri K. Masoumi S. M. Amini S. Goudarzi M. Tafreshi S. M. Bagheri A. (2023). Recent advances in metal nanoparticles to treat periodontitis. J. Nanobiotechnology 21, 283. 10.1186/s12951-023-02042-7 37605182 PMC10440939

[B170] Nes W. D. (2011). Biosynthesis of cholesterol and other sterols. Chem. Rev. 111, 6423–6451. 10.1021/cr200021m 21902244 PMC3191736

[B171] Neve A. Corrado A. Cantatore F. P. (2011). Osteoblast physiology in normal and pathological conditions. Cell Tissue Res. 343, 289–302. 10.1007/s00441-010-1086-1 21120535

[B172] Nguyen T. M. D. (2020). Adiponectin: role in physiology and pathophysiology. Int. J. Prev. Med. 11, 136. 10.4103/ijpvm.ijpvm_193_20 33088464 PMC7554603

[B173] Ning Z. Tan B. Chen B. Lau D. S. A. Wong T. M. Sun T. (2019). Precisely controlled delivery of abaloparatide through injectable hydrogel to promote bone regeneration. Macromol. Biosci. 19, e1900020. 10.1002/mabi.201900020 31066995

[B174] Nokhbehsaim M. Keser S. Nogueira A. V. B. Cirelli J. A. Jepsen S. Jäger A. (2014). Beneficial effects of adiponectin on periodontal ligament cells under normal and regenerative conditions. J. diabetes Res. 2014, 1–11. 10.1155/2014/796565 PMC4120919 25121107

[B175] Nowak K. M. Bodek K. H. Szterk A. Rudnicka K. Szymborski T. Kosieradzki M. (2019). Preclinical assessment of the potential of a 3D chitosan drug delivery system with sodium meloxicam for treating complications following tooth extraction. Int. J. Biol. Macromol. 133, 1019–1028. 10.1016/j.ijbiomac.2019.04.078 30986462

[B176] Nyan M. Sato D. Oda M. Machida T. Kobayashi H. Nakamura T. (2007). Bone formation with the combination of simvastatin and calcium sulfate in critical-sized rat calvarial defect. J. Pharmacol. Sci. 104, 384–386. 10.1254/jphs.sc0070184 17721043

[B177] O'Neill H. M. (2013). AMPK and exercise: glucose uptake and insulin sensitivity. Diabetes and metabolism J. 37, 1–21. 10.4093/dmj.2013.37.1.1 PMC3579147 23441028

[B178] Ortiz-Sánchez B. J. Legorreta-Herrera M. Rodriguez-Sosa M. J. M. O. I. (2021). Influence of gestational hormones on the bacteria‐induced cytokine response in periodontitis. Mediat. Inflamm. 2021, 1–12. 10.1155/2021/5834608 PMC8545568 34707462

[B179] Ortolani E. Guerriero M. Coli A. Di Giannuario A. Minniti G. Polimeni A. (2014). Effect of PDGF, IGF-1 and PRP on the implant osseointegration. An histological and immunohistochemical study in rabbits. Ann. Stomatol. 5, 66–68. PMC4071363 25002920

[B180] Ott S. M. Oleksik A. Lu Y. Harper K. Lips P. (2002). Bone histomorphometric and biochemical marker results of a 2‐year placebo‐controlled trial of raloxifene in postmenopausal women. J. bone mineral Res. 17, 341–348. 10.1359/jbmr.2002.17.2.341 11811565

[B181] Ouchi N. Kobayashi H. Kihara S. Kumada M. Sato K. Inoue T. (2004). Adiponectin stimulates angiogenesis by promoting cross-talk between AMP-activated protein kinase and Akt signaling in endothelial cells. J. Biol. Chem. 279, 1304–1309. 10.1074/jbc.m310389200 14557259 PMC4374490

[B182] Pacheco-Pantoja E. L. Alvarez-Nemegyei J. (2014). Statins and osteoporosis: a latent promise. Reumatol. Clin. 10, 201–203. 10.1016/j.reumae.2014.04.002 24882268

[B183] Padayatty S. J. Levine M. (2016). Vitamin C: the known and the unknown and Goldilocks. Oral Dis. 22, 463–493. 10.1111/odi.12446 26808119 PMC4959991

[B184] Padhy B. M. Gupta Y. K. (2011). Drug repositioning: re-investigating existing drugs for new therapeutic indications. J. Postgrad. Med. 57, 153–160. 10.4103/0022-3859.81870 21654146

[B185] Paglia D. N. Wey A. Breitbart E. A. Faiwiszewski J. Mehta S. K. Al-Zube L. (2013). Effects of local insulin delivery on subperiosteal angiogenesis and mineralized tissue formation during fracture healing. J. Orthop. Res. 31, 783–791. 10.1002/jor.22288 23238777 PMC6446235

[B186] Pan X. Lin X. Cao D. Zeng X. Yu P. S. He L. (2022). Deep learning for drug repurposing: methods, databases, and applications. WIREs Comput. Mol. Sci. 12, e1597. 10.1002/wcms.1597

[B187] Papapanou P. N. Susin C. (2017). Periodontitis epidemiology: is periodontitis under‐recognized, over‐diagnosed, or both? Periodontol. 2000 75, 45–51. 10.1111/prd.12200 28758302

[B188] Papi P. Di Carlo S. Rosella D. De Angelis F. Capogreco M. Pompa G. J. E. J. O. D. (2017). Peri-implantitis and extracellular matrix antibodies: a case–control study. Eur. J. Dent. 11, 340–344. 10.4103/ejd.ejd_28_17 28932144 PMC5594963

[B189] Park C. H. Oh J. H. Jung H. M. Choi Y. Rahman S. U. Kim S. (2017). Effects of the incorporation of ε-aminocaproic acid/chitosan particles to fibrin on cementoblast differentiation and cementum regeneration. Acta Biomater. 61, 134–143. 10.1016/j.actbio.2017.07.039 28764948

[B190] Park J. W. Kim J. M. Lee H. J. Jeong S. H. Suh J. Y. Hanawa T. (2014). Bone healing with oxytocin‐loaded microporous β‐TCPbone substitute in ectopic bone formation model and critical‐sized osseous defect of rat. J. Clin. Periodontol. 41, 181–190. 10.1111/jcpe.12198 24256613

[B191] Petersen P. E. Ogawa H. (2005). Strengthening the prevention of periodontal disease: the WHO approach. J. Periodontol. 76, 2187–2193. 10.1902/jop.2005.76.12.2187 16332229

[B192] Petit C. Batool F. Bugueno I. M. Schwinté P. Benkirane-Jessel N. Huck O. (2019). Contribution of statins towards periodontal treatment: a review. Mediat. Inflamm. 2019, 1–33. 10.1155/2019/6367402 PMC6415285 30936777

[B193] Pham D. T. Phewchan P. Navesit K. Chokamonsirikun A. Khemwong T. Tiyaboonchai W. (2021). Development of metronidazole-loaded *in situ* thermosensitive hydrogel for periodontitis treatment. Turk J. Pharm. Sci. 18, 510–516. 10.4274/tjps.galenos.2020.09623 34496558 PMC8430413

[B194] Pizzicannella J. Marconi G. D. Guarnieri S. Fonticoli L. Della Rocca Y. Konstantinidou F. (2021). Role of ascorbic acid in the regulation of epigenetic processes induced by Porphyromonas gingivalis in endothelial-committed oral stem cells. oral stem cells 156, 423–436. 10.1007/s00418-021-02014-8 PMC8604817 34370052

[B195] Pradeep A. R. Garg V. Kanoriya D. Singhal S. (2016a). 1.2% rosuvastatin versus 1.2% atorvastatin gel local drug delivery and redelivery in treatment of intrabony defects in chronic periodontitis: a randomized placebo-controlled clinical trial. J. Periodontology 87, 756–762. 10.1902/jop.2016.150706 26878748

[B196] Pradeep A. R. Garg V. Kanoriya D. Singhal S. (2016b). 1.2% rosuvastatin versus 1.2% atorvastatin gel local drug delivery and redelivery in treatment of intrabony defects in chronic periodontitis: a randomized placebo-controlled clinical trial. J. Periodontol. 87, 756–762. 10.1902/jop.2016.150706 26878748

[B197] Pradeep A. R. Rao N. S. Naik S. B. Kumari M. (2013). Efficacy of varying concentrations of subgingivally delivered metformin in the treatment of chronic periodontitis: a randomized controlled clinical trial. J. Periodontol. 84, 212–220. 10.1902/jop.2012.120025 22509750

[B198] Preshaw P. M. (2018). Host modulation therapy with anti‐inflammatory agents. Periodontol. 2000 76, 131–149. 10.1111/prd.12148 29193331

[B199] Pullar J. M. Carr A. C. Vissers M. (2017). The roles of vitamin C in skin health. Nutrients 9, 866. 10.3390/nu9080866 28805671 PMC5579659

[B200] Rabinovsky E. D. Draghia-Akli R. (2004). Insulin-like growth factor I plasmid therapy promotes *in vivo* angiogenesis. Mol. Ther. 9, 46–55. 10.1016/j.ymthe.2003.10.003 14741777

[B201] Ramanauskaite A. Schwarz F. Sader R. J. C. O. I. R. (2022). Influence of width of keratinized tissue on the prevalence of peri‐implant diseases: a systematic review and meta‐analysis. Clin. Oral Implants Res. 33, 8–31. 10.1111/clr.13766 35763022

[B202] Rams T. E. Slots J. (2023). Antimicrobial chemotherapy for recalcitrant severe human periodontitis. Antibiotics 12, 265. 10.3390/antibiotics12020265 36830176 PMC9951977

[B203] Ravishankar P. L. Kumar Y. P. Anila E. N. Chakraborty P. Malakar M. Mahalakshmi R. (2017). Effect of local application of curcumin and ornidazole gel in chronic periodontitis patients. Int. J. Pharm. Investig. 7, 188–192. 10.4103/jphi.jphi_82_17 PMC5903023 29692978

[B204] Reis R. (2019). Encyclopedia of tissue engineering and regenerative medicine. Academic Press.

[B205] Rejnmark L. Vestergaard P. Heickendorff L. Mosekilde L. (2010). Simvastatin does not affect vitamin d status, but low vitamin d levels are associated with dyslipidemia: results from a randomised, controlled trial. Int. J. Endocrinol. 2010, 1–6. 10.1155/2010/957174 PMC2778175 20016680

[B206] Rembe J.-D. Fromm-Dornieden C. Stuermer E. K. (2018). Effects of vitamin B complex and vitamin C on human skin cells: is the perceived effect measurable? Adv. skin and wound care 31, 225–233. 10.1097/01.asw.0000531351.85866.d9 29672394

[B207] Ren J. Fok M. R. Zhang Y. Han B. Lin Y. J. J. O. T. M. (2023). The role of non-steroidal anti-inflammatory drugs as adjuncts to periodontal treatment and in periodontal regeneration. J. Transl. Med. 21, 149. 10.1186/s12967-023-03990-2 36829232 PMC9960225

[B208] Ridolfo R. Tavakoli S. Junnuthula V. Williams D. S. Urtti A. Van Hest J. C. J. B. (2020). Exploring the impact of morphology on the properties of biodegradable nanoparticles and their diffusion in complex biological medium. Biomacromolecules 22, 126–133. 10.1021/acs.biomac.0c00726 32510218 PMC7805011

[B209] Rocha-García D. Guerra-Contreras A. Rosales-Mendoza S. Palestino G. J. O. M. S. (2016). Role of porous silicon/hydrogel composites on drug delivery. Open Mater. Sci. 3, 10.1515/mesbi-2016-0011

[B210] Rodríguez-Carballo E. Gámez B. Ventura F. (2016). p38 MAPK signaling in osteoblast differentiation. Front. Cell Dev. Biol. 4, 40. 10.3389/fcell.2016.00040 27200351 PMC4858538

[B211] Rokaya D. Srimaneepong V. Wisitrasameewon W. Humagain M. Thunyakitpisal P. J. E. J. O. D. (2020). Peri-implantitis update: risk indicators, diagnosis, and treatment. diagnosis, Treat. 14, 672–682. 10.1055/s-0040-1715779 PMC7536094 32882741

[B212] Sadek K. M. Shib N. A. Taher E. S. Rashed F. Shukry M. Atia G. A. (2024). Harnessing the power of bee venom for therapeutic and regenerative medical applications: an updated review. Front. Pharmacol. 15, 1412245. 10.3389/fphar.2024.1412245 39092234 PMC11291246

[B213] Safari B. Davaran S. Aghanejad A. (2021). Osteogenic potential of the growth factors and bioactive molecules in bone regeneration. Int. J. Biol. Macromol. 175, 544–557. 10.1016/j.ijbiomac.2021.02.052 33571587

[B214] Saleem M. J. P. J. O. S. 2021. Aloe barbadensis miller: a comprehensive review. Pak. J. Sci. 73 (4). 684–691. 10.57041/pjs.v73i4.388

[B215] Santos C. F. D. Akashi A. E. Dionísio T. J. Sipert C. R. Didier D. N. Greene A. S. (2009). Characterization of a local renin‐angiotensin system in rat gingival tissue. J. periodontology 80, 130–139. 10.1902/jop.2009.080264 PMC2758819 19228099

[B216] Saravi B. E. Lang G. Ülkümen S. Burchard T. Weihrauch V. Patzelt S. (2020). The tissue renin-angiotensin system (tRAS) and the impact of its inhibition on inflammation and bone loss in the periodontal tissue. Eur. Cell. Mater. 40, 203–226. 10.22203/ecm.v040a13 33170502

[B217] Satny M. Hubacek J. A. Vrablik M. (2021). Statins and inflammation. Curr. Atheroscler. Rep. 23, 80–88. 10.1007/s11883-021-00977-6 34851454

[B218] Sattary M. Rafienia M. Kazemi M. Salehi H. Mahmoudzadeh M. (2019). Promoting effect of nano hydroxyapatite and vitamin D3 on the osteogenic differentiation of human adipose-derived stem cells in polycaprolactone/gelatin scaffold for bone tissue engineering. Mater. Sci. Eng. C 97, 141–155. 10.1016/j.msec.2018.12.030 30678899

[B219] Shafiei -Irannejad V. Samadi N. Salehi R. Yousefi B. Zarghami N. (2017). New insights into antidiabetic drugs: possible applications in cancer treatment. Chem. Biol. Drug Des. 90, 1056–1066. 10.1111/cbdd.13013 28456998

[B220] Shetty S. Thomas B. Shetty V. Bhandary R. Shetty R. M. J. A. (2013). An *in-vitro* evaluation of the efficacy of garlic extract as an antimicrobial agent on periodontal pathogens: a microbiological study. Ayu 34, 445–451. 10.4103/0974-8520.127732 24695825 PMC3968712

[B221] Shino H. Hasuike A. Arai Y. Honda M. Isokawa K. Sato S. (2016). Melatonin enhances vertical bone augmentation in rat calvaria secluded spaces. Med. oral, Patol. oral cirugia bucal 21, e122–e126. 10.4317/medoral.20904 PMC4765744 26595835

[B222] Sholapurkar A. Sharma D. Glass B. Miller C. Nimmo A. Jennings E. J. D. J. (2020). Professionally delivered local antimicrobials in the treatment of patients with periodontitis—a narrative review. Dent. J. (Basel). 9, 2. 10.3390/dj9010002 33375176 PMC7822216

[B223] Singh K. D. Karnik S. S. (2016). Angiotensin receptors: structure, function, signaling and clinical applications. J. Cell Signal 1, 111. 10.4172/jcs.1000111 27512731 PMC4976824

[B224] Siqueira C. R. B. D. Semenoff T. A. D. V. Palma V. C. Borges Á. H. Silva N. F. D. Segundo A. S. (2015). Effect of chronic stress on implant osseointegration into rat's mandible. Acta Cirúrgica Bras. 30, 598–603. 10.1590/s0102-865020150090000003 26465103

[B225] Sirakov M. Skah S. Nadjar J. Plateroti M. (2013). Thyroid hormone's action on progenitor/stem cell biology: new challenge for a classic hormone? Biochimica Biophysica Acta (BBA)-General Subj. 1830, 3917–3927. 10.1016/j.bbagen.2012.07.014 22890105

[B226] Song C. Wang J. Song Q. Li X. Chen Z. Ma Q. (2008). Simvastatin induces estrogen receptor-alpha (ER-α) in murine bone marrow stromal cells. J. Bone Min. Metab. 26, 213–217. 10.1007/s00774-007-0820-6 18470660

[B227] Sonnenschein S. K. Meyle J. (2015). Local inflammatory reactions in patients with diabetes and periodontitis. Periodontol. 2000 69, 221–254. 10.1111/prd.12089 26252411

[B228] Sotelo-Barrera M. Cília-García M. Luna-Cavazos M. Díaz-Núñez J. L. Romero-Manzanares A. Soto-Hernández R. M. (2022). Amphipterygium adstringens (schltdl.) schiede ex standl (anacardiaceae): an endemic plant with relevant pharmacological properties. Plants (Basel). 11, 1766. 10.3390/plants11131766 35807718 PMC9268796

[B229] Spiro A. S. Khadem S. Jeschke A. Marshall R. P. Pogoda P. Ignatius A. (2013). The SERM raloxifene improves diaphyseal fracture healing in mice. J. bone mineral metabolism 31, 629–636. 10.1007/s00774-013-0461-x 23546819

[B230] Stańdo M. Piatek P. Namiecinska M. Lewkowicz P. Lewkowicz N. (2020). Omega-3 polyunsaturated fatty acids EPA and DHA as an adjunct to non-surgical treatment of periodontitis: a randomized clinical trial. Nutrients 12, 2614. 10.3390/nu12092614 32867199 PMC7551834

[B231] Stefanaki C. Pervanidou P. Boschiero D. Chrousos G. P. (2018). Chronic stress and body composition disorders: implications for health and disease. Hormones 17, 33–43. 10.1007/s42000-018-0023-7 29858868

[B232] Stutz C. Batool F. Petit C. Strub M. Kuchler-Bopp S. Benkirane-Jessel N. (2020). Influence of parathyroid hormone on periodontal healing in animal models: a systematic review. A Syst. Rev. 120, 104932. 10.1016/j.archoralbio.2020.104932 33113458

[B233] Sun P. Wang M. Yin G.-Y. (2020). Endogenous parathyroid hormone (PTH) signals through osteoblasts via RANKL during fracture healing to affect osteoclasts. Biochem. Biophysical Res. Commun. 525, 850–856. 10.1016/j.bbrc.2020.02.177 32169280

[B234] Sun Y. Li J. Xie X. Gu F. Sui Z. Zhang K. (2021). Macrophage-osteoclast associations: origin, polarization, and subgroups. Front. Immunol. 12, 778078. 10.3389/fimmu.2021.778078 34925351 PMC8672114

[B235] Suriyagoda L. Mohotti A. J. Vidanarachchi J. K. Kodithuwakku S. P. Chathurika M. Bandaranayake P. C. (2021). “Ceylon cinnamon”: much more than just a spice. Plants People Planet 3, 319–336. 10.1002/ppp3.10192

[B236] Swanson W. B. Yao Y. Mishina Y. J. G. (2022). Novel approaches for periodontal tissue engineering. Genesis 60, e23499. 10.1002/dvg.23499 36086991 PMC9787372

[B237] Taalab M. R. Rehim S. S. A. E. Eldeeb D. W. El-Moslemany R. M. Abdelrahman H. J. S. R. (2023). Histologic and histomorphometric evaluation of Aloe vera adjunctive to β-tricalcium phosphate in class II furcation defects in dogs. Sci. Rep. 13, 4198. 10.1038/s41598-023-31282-8 36918622 PMC10015024

[B238] Tai I. C. Fu Y. C. Wang C. K. Chang J. K. Ho M. L. (2013). Local delivery of controlled-release simvastatin/PLGA/HAp microspheres enhances bone repair. Int. J. Nanomedicine 8, 3895–3904. 10.2147/ijn.s48694 24143094 PMC3798145

[B239] Tallawi M. Rosellini E. Barbani N. Cascone M. G. Rai R. Saint-Pierre G. (2015). Strategies for the chemical and biological functionalization of scaffolds for cardiac tissue engineering: a review. J. R. Soc. Interface 12, 20150254. 10.1098/rsif.2015.0254 26109634 PMC4528590

[B240] Tanabe K. Nomoto H. Okumori N. Miura T. Yoshinari M. (2012). Osteogenic effect of fluvastatin combined with biodegradable gelatin-hydrogel. Dent. Mater J. 31, 489–493. 10.4012/dmj.2012-008 22673476

[B241] Tang G.-Y. Meng X. Gan R.-Y. Zhao C.-N. Liu Q. Feng Y.-B. (2019). Health functions and related molecular mechanisms of tea components: an update review. Int. J. Mol. Sci. 20, 6196. 10.3390/ijms20246196 31817990 PMC6941079

[B242] Taskan M. M. Gevrek F. (2020). PPAR-γ, RXR, VDR, and COX-2 Expressions in gingival tissue samples of healthy individuals, periodontitis and peri-implantitis patients. Niger. J. Clin. Pract. 23, 46–53. 10.4103/njcp.njcp_349_19 31929206

[B243] Taymour N. Haque M. A. Atia G. A. N. Mohamed S. Z. Rokaya D. Bajunaid S. M. (2024). Nanodiamond: a promising carbon-based nanomaterial for therapeutic and regenerative dental applications. ChemistrySelect 9, e202401328. 10.1002/slct.202401328

[B244] Terruzzi I. Montesano A. Senesi P. Villa I. Ferraretto A. Bottani M. (2019). L-carnitine reduces oxidative stress and promotes cells differentiation and bone matrix proteins expression in human osteoblast-like cells. Biomed. Res. Int. 2019, 1–13. 10.1155/2019/5678548 PMC6360619 30800672

[B245] Thomas D. M. Udagawa N. Hards D. K. Quinn J. M. Moseley J. M. Findlay D. M. (1998). Insulin receptor expression in primary and cultured osteoclast-like cells. Bone 23, 181–186. 10.1016/s8756-3282(98)00095-7 9737339

[B246] Toraman A. Arabaci T. Aytekin Z. Albayrak M. Bayir Y. (2020). Effects of vitamin C local application on ligature-induced periodontitis in diabetic rats. J. Appl. Oral Sci. 28, e20200444. 10.1590/1678-7757-2020-0444 33263670 PMC7695129

[B247] Torres-Ortiz D. García-Alcocer G. Loske A. M. Fernández F. Becerra-Becerra E. Esparza R. (2023). Green synthesis and antiproliferative activity of gold nanoparticles of a controlled size and shape obtained using shock wave extracts from amphipterygium adstringens. Bioeng. (Basel). 10, 437. 10.3390/bioengineering10040437 PMC10136038 37106624

[B248] Trindade D. Carvalho R. Machado V. Chambrone L. Mendes J. J. Botelho J. J. J. O. C. P. (2023). Prevalence of periodontitis in dentate people between 2011 and 2020: a systematic review and meta-analysis of epidemiological studies. J. Clin. Periodontol. 50, 604–626. 10.1111/jcpe.13769 36631982

[B249] Trucillo P. J. P. (2022). Drug carriers: a review on the most used mathematical models for drug release. Process. (Basel). 10, 1094. 10.3390/pr10061094

[B250] Urano T. Shiraki M. Kuroda T. Tanaka S. Uenishi K. Inoue S. (2017). Preventive effects of raloxifene treatment on agerelated weight loss in postmenopausal women. J. bone mineral metabolism 35, 108–113. 10.1007/s00774-015-0733-8 26754796

[B251] Vaidya B. Parvathaneni V. Kulkarni N. S. Shukla S. K. Damon J. K. Sarode A. (2019). Cyclodextrin modified erlotinib loaded PLGA nanoparticles for improved therapeutic efficacy against non-small cell lung cancer. Int. J. Biol. Macromol. 122, 338–347. 10.1016/j.ijbiomac.2018.10.181 30401652

[B252] Van De Ven C. J. J. M. Bakker N. E. C. Link D. P. Geven E. J. W. Gossen J. A. (2021a). Sustained release of ancillary amounts of testosterone and alendronate from PLGA coated pericard membranes and implants to improve bone healing. Plos one 16, e0251864. 10.1371/journal.pone.0251864 33999955 PMC8128250

[B253] Van De Ven C. Bakker N. E. C. Link D. P. Geven E. J. W. Gossen J. A. (2021b). Sustained release of ancillary amounts of testosterone and alendronate from PLGA coated pericard membranes and implants to improve bone healing. PLoS One 16, e0251864. 10.1371/journal.pone.0251864 33999955 PMC8128250

[B254] Vanea E. Moraru C. Vulpoi A. Cavalu S. Simon V. (2014). Freeze-dried and spray-dried zinc-containing silica microparticles entrapping insulin. J. Biomater. Appl. 28, 1190–1199. 10.1177/0885328213501216 23985534

[B255] Viglianisi G. Santonocito S. Lupi S. M. Amato M. Spagnuolo G. Pesce P. (2023). Impact of local drug delivery and natural agents as new target strategies against periodontitis: new challenges for personalized therapeutic approach. Ther. Adv. Chronic Dis. 14. 10.1177/20406223231191043 PMC10501082 37720593

[B256] Visser R. Rico-Llanos G. A. Pulkkinen H. Becerra J. (2016). Peptides for bone tissue engineering. J. Control Release 244, 122–135. 10.1016/j.jconrel.2016.10.024 27794492

[B257] Wada-Mihara C. Seto H. Ohba H. Tokunaga K. Kido J. I. Nagata T. (2018). Local administration of calcitonin inhibits alveolar bone loss in an experimental periodontitis in rats. Biomed. Pharmacother. 97, 765–770. 10.1016/j.biopha.2017.10.165 29107933

[B258] Wanat K. J. M. B. R. (2020). Biological barriers, and the influence of protein binding on the passage of drugs across them. Mol. Biol. Rep. 47, 3221–3231. 10.1007/s11033-020-05361-2 32140957

[B259] Wang Y.-G. Qu X.-H. Yang Y. Han X.-G. Wang L. Qiao H. (2016). AMPK promotes osteogenesis and inhibits adipogenesis through AMPK-Gfi1-OPN axis. Cell. Signal. 28, 1270–1282. 10.1016/j.cellsig.2016.06.004 27283242

[B260] Wang D. Steffi C. Wang Z. Kong C. H. Lim P. N. Shi Z. (2018a). Beta-cyclodextrin modified mesoporous bioactive glass nanoparticles/silk fibroin hybrid nanofibers as an implantable estradiol delivery system for the potential treatment of osteoporosis. Nanoscale 10, 18341–18353. 10.1039/c8nr05268a 30255905

[B261] Wang J. Su B. Jiang H. Cui N. Yu Z. Yang Y. (2020a). Traditional uses, phytochemistry and pharmacological activities of the genus Cinnamomum (Lauraceae): a review. A Rev. 146, 104675. 10.1016/j.fitote.2020.104675 32561421

[B262] Wang N. Cheng X. Li N. Wang H. Chen H. J. A. H. M. (2019). Nanocarriers and their loading strategies. Adv. Healthc. Mater. 8, 1801002. 10.1002/adhm.201801002 30450761

[B263] Wang Q. Zhou X. Zhang P. Zhao P. Nie L. Ji N. (2020b). 25-Hydroxyvitamin D3 positively regulates periodontal inflammaging via SOCS3/STAT signaling in diabetic mice. Steroids 156, 108570. 10.1016/j.steroids.2019.108570 31917967

[B264] Wang T. Ye J. Zhang Y. Li J. Yang T. Wang Y. (2024). Role of oxytocin in bone. Front. Endocrinol. (Lausanne). 15, 1450007. 10.3389/fendo.2024.1450007 39290327 PMC11405241

[B265] Wang X. Zhang G. Qi F. Cheng Y. Lu X. Wang L. (2018b). Enhanced bone regeneration using an insulin-loaded nano-hydroxyapatite/collagen/PLGA composite scaffold. Int. J. Nanomedicine 13, 117–127. 10.2147/ijn.s150818 PMC5743129 29317820

[B266] Wang Y. Harrington P. D. B. Chen P. J. A. Chemistry B. (2020c). Metabolomic profiling and comparison of major cinnamon species using UHPLC–HRMS. Anal. Bioanal. Chem. 412, 7669–7681. 10.1007/s00216-020-02904-1 32875369 PMC7541615

[B267] Wang Y. Zhang Y. Shi Y.-Q. Pan X.-H. Lu Y.-H. Cao P. J. M. P. (2018c). Antibacterial effects of cinnamon (Cinnamomum zeylanicum) bark essential oil on Porphyromonas gingivalis. Microb. Pathog. 116, 26–32. 10.1016/j.micpath.2018.01.009 29325862

[B268] Wang Z. Chen Z. Fang F. Qiu W. (2021). The role of adiponectin in periodontitis: current state and future prospects. Biomed. and Pharmacother. 137, 111358. 10.1016/j.biopha.2021.111358 33561644

[B269] Wan Hasan W. N. Chin K.-Y. Abd Ghafar N. Soelaiman I. N. J. D. D. (2020). Annatto-derived tocotrienol promotes mineralization of MC3T3-E1 cells by enhancing BMP-2 protein expression via inhibiting RhoA activation and HMG-CoA reductase gene expression. Drug Des. devel. Ther., 14, 969–976. 10.2147/dddt.s224941 PMC7060796 32184566

[B270] Warren K. R. Postolache T. T. Groer M. E. Pinjari O. Kelly D. L. Reynolds M. A. (2014). Role of chronic stress and depression in periodontal diseases. Periodontol. 2000 64, 127–138. 10.1111/prd.12036 24320960 PMC7167640

[B271] Wein M. N. Kronenberg H. M. (2018). Regulation of bone remodeling by parathyroid hormone. Cold Spring Harb. Perspect. Med. 8, a031237. 10.1101/cshperspect.a031237 29358318 PMC6071549

[B272] Weivoda M. M. Hohl R. J. (2011). Effects of farnesyl pyrophosphate accumulation on calvarial osteoblast differentiation. Endocrinology 152, 3113–3122. 10.1210/en.2011-0016 21586555

[B273] Wojda S. J. Donahue S. W. (2018). Parathyroid hormone for bone regeneration. J. Orthop. Res. 36, 2586–2594. 10.1002/jor.24075 29926970

[B274] Wong R. W. Rabie A. B. (2003). Statin collagen grafts used to repair defects in the parietal bone of rabbits. Br. J. Oral Maxillofac. Surg. 41, 244–248. 10.1016/s0266-4356(03)00081-0 12946667

[B275] Wu S.-C. Chang C.-H. Chang L.-H. Wu C.-W. Chen J.-W. Chen C.-H. (2021). Simvastatin enhances the chondrogenesis but not the osteogenesis of adipose-derived stem cells in a hyaluronan microenvironment. Biomedicines. 9, 559. 10.3390/biomedicines9050559 34067739 PMC8156330

[B276] Wu L. Luo Z. Liu Y. Jia L. Jiang Y. Du J. (2019). Aspirin inhibits RANKL-induced osteoclast differentiation in dendritic cells by suppressing NF-κB and NFATc1 activation. Stem Cell Res. Ther. 10, 375. 10.1186/s13287-019-1500-x 31805984 PMC6894480

[B277] Xue W. Yu J. Chen W. J. B. R. I. (2018). Plants and their bioactive constituents in mesenchymal stem cell-based periodontal regeneration: a novel prospective. Biomed. Res. Int. 2018, 1–15. 10.1155/2018/7571363 PMC6098897 30175141

[B278] Xu X. Burgess D. J. (2012). “Liposomes as carriers for controlled drug delivery,” in Long acting injections and implants. Springer.

[B279] Xu X. Gu Z. Chen X. Shi C. Liu C. Liu M. (2019a). An injectable and thermosensitive hydrogel: promoting periodontal regeneration by controlled-release of aspirin and erythropoietin. Acta Biomater. 86, 235–246. 10.1016/j.actbio.2019.01.001 30611793

[B280] Xu X. Gu Z. Chen X. Shi C. Liu C. Liu M. (2019b). An injectable and thermosensitive hydrogel: promoting periodontal regeneration by controlled-release of aspirin and erythropoietin. Acta Biomater. 86, 235–246. 10.1016/j.actbio.2019.01.001 30611793

[B281] Yang F. Cho S.-W. Son S. M. Bogatyrev S. R. Singh D. Green J. J. (2010). Genetic engineering of human stem cells for enhanced angiogenesis using biodegradable polymeric nanoparticles. Proc. Natl. Acad. Sci. U. S. A. 107, 3317–3322. 10.1073/pnas.0905432106 19805054 PMC2840438

[B282] Yang S. Liu H. Liu Y. Liu L. Zhang W. Luo E. (2019). Effect of adiponectin secreted from adipose‐derived stem cells on bone‐fat balance and bone defect healing. J. tissue Eng. Regen. Med. 13, 2055–2066. 10.1002/term.2915 31210408

[B283] Yar M. Farooq A. Shahzadi L. Khan A. S. Mahmood N. Rauf A. (2016a). Novel meloxicam releasing electrospun polymer/ceramic reinforced biodegradable membranes for periodontal regeneration applications. Mater. Sci. Eng. C 64, 148–156. 10.1016/j.msec.2016.03.072 27127039

[B284] Yar M. Farooq A. Shahzadi L. Khan A. S. Mahmood N. Rauf A. (2016b). Novel meloxicam releasing electrospun polymer/ceramic reinforced biodegradable membranes for periodontal regeneration applications. Mater Sci. Eng. C Mater Biol. Appl. 64, 148–156. 10.1016/j.msec.2016.03.072 27127039

[B285] Yavuz B. Ertugrul D. T. Cil H. Ata N. Akin K. O. Yalcin A. A. (2009). Increased levels of 25 hydroxyvitamin D and 1,25-dihydroxyvitamin D after rosuvastatin treatment: a novel pleiotropic effect of statins? Cardiovasc Drugs Ther. 23, 295–299. 10.1007/s10557-009-6181-8 19543962

[B286] Ye K. Kuang H. You Z. Morsi Y. Mo X. J. P. (2019). Electrospun nanofibers for tissue engineering with drug loading and release. Pharmaceutics 11, 182. 10.3390/pharmaceutics11040182 30991742 PMC6523318

[B287] Yong J. Von Bremen J. Ruiz-Heiland G. Ruf S. (2020). Adiponectin interacts in-vitro with cementoblasts influencing cell migration, proliferation and cementogenesis partly through the MAPK signaling pathway. Front. Pharmacol. 11, 585346. 10.3389/fphar.2020.585346 33414717 PMC7783624

[B288] Yong J. Von Bremen J. Ruiz-Heiland G. Ruf S. (2021). Adiponectin as well as compressive forces regulate *in vitro* β-catenin expression on cementoblasts via mitogen-activated protein kinase signaling activation. Front. Cell Dev. Biol. 9, 645005. 10.3389/fcell.2021.645005 33996803 PMC8113767

[B289] Yoshii T. Hafeman A. E. Nyman J. S. Esparza J. M. Shinomiya K. Spengler D. M. (2010). A sustained release of lovastatin from biodegradable, elastomeric polyurethane scaffolds for enhanced bone regeneration. Tissue Eng. Part A 16, 2369–2379. 10.1089/ten.tea.2009.0585 20205517

[B290] Yousefi L. Leylabadlo H. E. Pourlak T. Eslami H. Taghizadeh S. Ganbarov K. (2020). Oral spirochetes: pathogenic mechanisms in periodontal disease. Microb. Pathog. 144, 104193. 10.1016/j.micpath.2020.104193 32304795

[B291] Yousuf D. A. Afify O. M. El Soudany K. S. Ghoniem S. M. (2013). The effect of local application of melatonin gel on the healing of periodontal osseous defects in experimentally induced diabetes in rabbits. Tanta Dent. J. 10, 48–57. 10.1016/j.tdj.2013.08.003

[B292] Yussif N. M. Abdul Aziz M. A. Abdel Rahman A. R. (2016). Evaluation of the anti-inflammatory effect of locally delivered vitamin C in the treatment of persistent gingival inflammation: clinical and histopathological study. J. Nutr. Metab. 2016, 2978741–2978748. 10.1155/2016/2978741 28050280 PMC5165168

[B293] Zhang M.-L. Cheng J. Xiao Y.-C. Yin R.-F. Feng X. (2017). Raloxifene microsphere-embedded collagen/chitosan/β-tricalcium phosphate scaffold for effective bone tissue engineering. Int. J. Pharm. 518, 80–85. 10.1016/j.ijpharm.2016.12.031 27988379

[B294] Zhang L. Li X. Yan H. Huang L. J. M. (2018). Salivary matrix metalloproteinase (MMP)-8 as a biomarker for periodontitis: a PRISMA-compliant systematic review and meta-analysis. Med. Baltim. 97, e9642. 10.1097/md.0000000000009642 PMC5779768 29504999

[B295] Zhang M. Yang B. Peng S. Xiao J. J. S. C. (2021). Metformin rescues the impaired osteogenesis differentiation ability of rat adipose-derived stem cells in high glucose by activating autophagy. Stem Cells Dev. 30, 1017–1027. 10.1089/scd.2021.0181 34486387

[B296] Zhang X. Hasani-Sadrabadi M. M. Zarubova J. Dashtimighadam E. Haghniaz R. Khademhosseini A. (2022a). Immunomodulatory microneedle patch for periodontal tissue regeneration. Matter 5, 666–682. 10.1016/j.matt.2021.11.017 35340559 PMC8942382

[B297] Zhang Y. Ding N. Zhang T. Sun Q. Han B. Yu T. (2019). A tetra-PEG hydrogel based aspirin sustained release system exerts beneficial effects on periodontal ligament stem cells mediated bone regeneration. Front. Chem. 7, 682. 10.3389/fchem.2019.00682 31681732 PMC6811605

[B298] Zhang Y. Dou X. Zhang L. Wang H. Zhang T. Bai R. (2022b). Facile fabrication of a biocompatible composite gel with sustained release of aspirin for bone regeneration. Bioact. Mater 11, 130–139. 10.1016/j.bioactmat.2021.09.033 34938918 PMC8665342

[B299] Zhang Z. Xu W. Zhang Z. Chen X. Jin H. Jiang N. (2024). The bone-protective benefits of kaempferol combined with metformin by regulation of osteogenesis-angiogenesis coupling in OVX rats. Biomed. Pharmacother. 173 **,** 116364. 10.1016/j.biopha.2024.116364 38447449

[B300] Zhong X. Xiu L. L. Wei G. H. Liu Y. Y. Su L. Cao X. P. (2011). Bezafibrate enhances proliferation and differentiation of osteoblastic MC3T3-E1 cells via AMPK and eNOS activation. Acta Pharmacol. Sin. 32, 591–600. 10.1038/aps.2011.15 21499286 PMC4002510

[B301] Zhou W. L. Li L. L. Qiu X. R. An Q. Li M. H. (2017). Effects of combining insulin-like growth factor 1 and platelet-derived growth factor on osteogenesis around dental implants. Chin. J. Dent. Res. 20, 105–109. 10.3290/j.cjdr.a38275 28573264

[B302] Zhou X. Liu P. Nie W. Peng C. Li T. Qiang L. (2020). Incorporation of dexamethasone-loaded mesoporous silica nanoparticles into mineralized porous biocomposite scaffolds for improving osteogenic activity. Int. J. Biol. Macromol. 149, 116–126. 10.1016/j.ijbiomac.2020.01.237 31987948

[B303] Zupancic S. Sinha-Ray S. Sinha-Ray S. Kristl J. Yarin A. L. J. M. P. (2016). Long-term sustained ciprofloxacin release from PMMA and hydrophilic polymer blended nanofibers. Mol. Pharm. 13, 295–305. 10.1021/acs.molpharmaceut.5b00804 26635214

