# Supplementary figures and images for: Prospective and challenges of locally applied repurposed pharmaceuticals for periodontal tissue regeneration
Source: Front Bioeng Biotechnol. 2024 Nov 13;12:1400472. doi: 10.3389/fbioe.2024.1400472 (PMC11600316; doi:10.3389/fbioe.2024.1400472)

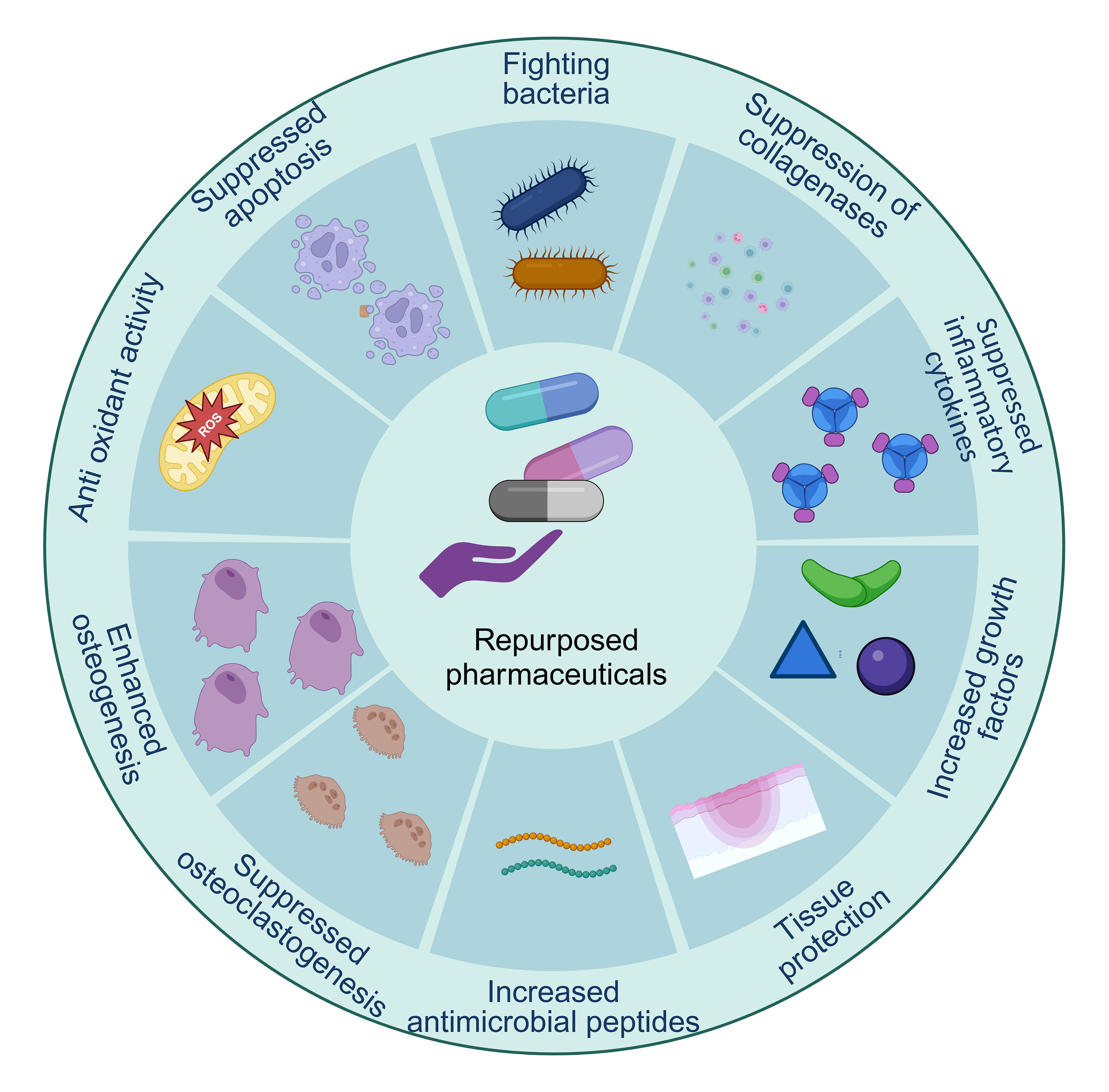

Supplement: Supplementary file 1 [file Image1.JPEG]
